# Investigation of Flavor and Functional Properties of Diverse Yellow Pea Ingredients for Pan Bread Applications

**DOI:** 10.1111/1750-3841.70724

**Published:** 2025-12-11

**Authors:** Alexandre D. Goertzen, Donna Ryland, Shiva Shariati‐Ievari, Karen Pitura, Lindsay Bourré, Praiya Asavajaru, Nandhakishore Rajagopalan, Anusha G. P. Samaranayaka, Brittany Polley, Pankaj Bhowmik, Michel Aliani

**Affiliations:** ^1^ Department of Food and Human Nutritional Sciences University of Manitoba Winnipeg Manitoba Canada; ^2^ Division of Neurodegenerative and Neurodevelopmental Disorders St. Boniface Hospital Albrechtsen Research Centre Winnipeg Manitoba Canada; ^3^ Cereals Canada Winnipeg Manitoba Canada; ^4^ Aquatic and Crop Resource Development Centre National Research Council Canada Saskatoon Saskatchewan Canada; ^5^ Department of Chemical and Biological Engineering University of Saskatchewan Saskatoon Saskatchewan Canada; ^6^ Industrial Research Assistance Program National Research Council Canada Saskatoon Saskatchewan Canada

**Keywords:** electronic nose, flavoromics, heat‐treated yellow pea flour, infrared radiation, lipoxygenase (LOX), pea protein isolate and concentrate, radiofrequency radiation, sensory evaluation, volatile organic compounds (VOCs)

## Abstract

Yellow peas (YPs) are a promising source of sustainable plant proteins however, characteristic off‐flavors limit consumer acceptance and hinder their use as value‐added food ingredients. This study evaluated the compositional and sensory characteristics of fourteen processed YP ingredients to determine their suitability in pan breads. Nine protein isolates (YPIs), three protein concentrates (YPCs), and two heat‐treated flours (YP‐IR and YP‐RF) were analyzed for fatty acid composition, lipoxygenase (LOX) activity, volatile organic compounds (VOCs), and electronic nose (eNose) responses. LOX activity and linoleic acid, a key LOX substrate, differed significantly between YP ingredients. Fifteen VOCs previously linked to off‐flavors were identified, with YPIs exhibiting the highest concentrations. eNose analysis correctly predicted YP ingredient identities with 69.5% accuracy and distinguished treated flours from other YP ingredients. Pan breads using two YPI ingredients (YPI‐1 and YPI‐2) predicted to produce strong off‐flavors, the two heat‐treated flours and a wheat flour (WF) control were prepared. Breads were evaluated for proximate composition, consumer acceptance, descriptive sensory attributes, and instrumental color. YPI‐1 bread was significantly less acceptable overall than WF (*p* < 0.001) and showed higher pea aroma and flavor (*p* < 0.001). YPI‐2 bread had a denser texture (*p* < 0.05) and significantly higher wheaty aroma (*p* < 0.01). Breads made with YP‐IR and YP‐RF performed closest to the WF control. The heat‐treated YP flours demonstrated the greatest potential as pan bread additives. YPI samples were associated with undesirable sensory characteristics. Analyses of the raw YP ingredients effectively predicted sensory differences, highlighting the usefulness of these methods as screening tools during product development.

**Practical Applications:**

This research shows that different value‐added yellow pea ingredients can introduce a range of flavors into food matrices, and that flavoromics tools can assist with product development. The two heat‐treated yellow pea flours were identified as the best options for bread making because they were less related to pea aroma and flavor in the final product. These findings can help bakers and food manufacturers choose pea ingredients that make high‐protein breads taste better.

## 1 Introduction

From 2012 to 2021, production of peas (*Pisum sativum*), including both green and yellow pea (YP) varieties, averaged 3.784 million tons per year in Western Canada, accounting for roughly one‐quarter to one‐third of global production over the same period (Canadian Grain Commision 2022). These crops are notable for producing high levels of protein, being gluten‐free, and having an amino acid profile complementary to that of cereals, as well as micronutrients including selenium, thiamin, niacin, folate, and potassium (Bresciani and Marti 2019). The incorporation of peas in crop rotations also supports sustainable agricultural practices as their drought resistance and associations with nitrogen‐fixing bacteria lessen dependence on external agricultural inputs (Crews and Peoples 2004). Finally, pea proteins have good functional properties, including high solubility, water‐ and oil‐holding capacity, and emulsion forming capacity, making them suitable for incorporation into a wide range of foods (Shanthakumar et al. 2022).

These properties have made peas a focus for the development of value‐added ingredients, and several producers now offer protein‐enriched pea products such as protein concentrates and protein isolates, as well as various treated flours (Shanthakumar et al. 2022). These materials are being increasingly utilized in modern food applications, from plant‐based meat alternatives to protein‐enriched beverages and snacks, and to fortify bakery products like breads and biscuits. Nevertheless, pea‐derived ingredients can face consumer acceptance challenges due to the presence of undesirable sensory notes, commonly described as “bitter,” “sweet,” “pea‐like/beany,” “cardboard‐like,” “grassy,” “grainy,” and “earthy” flavors and aromas (Wang et al. 2022).

These sensory characteristics originate from volatile organic compounds (VOCs) which are released from pea materials during consumption, and then detected by the human olfactory system (Viana and English 2021). Although peas contain a wide range of VOCs, previous research has identified only a subset of these compounds as major sources of off‐flavors. For instance, several methoxy‐pyrazine compounds have been identified with “vegetable” or “pea‐like” aromas (Murray et al. 1976), whereas several aldehydes including hexanal, heptanal, and furan‐2‐pentyl have been identified with the presence of “grassy” and “green” off‐flavors (Bhowmik et al. 2023). Some of these compounds are also byproducts of lipoxygenase (LOX)‐catalyzed degradation of linoleic and α‐linolenic acids (Viana and English 2021), reactions that occur in pea materials both pre‐ and postharvest and which are considered a major source of off‐flavors (Fahmi et al. 2019).

As a result, some researchers have focused on developing pea cultivars with reduced LOX activity (Bhowmik et al. 2023). Postharvest LOX activity, can also be reduced by heat treatment (Asavajaru et al. 2025), which can provide additional benefits such as decreasing microbial activity and modifying functional and nutritional properties (Bermudez‐Aguirre and Niemira 2023). To perform these treatments, direct irradiation techniques, including radiofrequency (RF) and infrared (IR) heating, are becoming increasingly popular as they are generally more energy efficient and allow for more uniform heating compared to traditional methods (Das et al. 2025). However, these irradiative techniques do differ in their exact mechanisms, which may influence the properties of their final products. RF heating causes small polar molecules, especially water, to rapidly rotate in an alternating electric field, generating frictional heating in a food matrix. In contrast, IR heating transfers energy, depending on the exact frequency used, to molecules with specific chemical bonds, particularly O─H, C ═ O, C ═ C, and N─H (Yadav et al. 2020).

Other physical and chemical treatments can also reduce LOX activity or otherwise alter the VOC contents of YP materials. For instance, fermentation has been used to degrade off‐flavor compounds and reduce relevant off‐flavor characteristics in pea materials (El Youssef et al. 2020). Alkaline extraction followed by isoelectric precipitation, a common method for producing protein isolates, can similarly remove certain VOCs and also denature enzymes (Shanthakumar et al. 2022). Furthermore, this method usually follows pretreatments that aim to eliminate fatty acids, including LOX enzyme substrates. In contrast, the “dry‐fractionation” techniques used to produce protein concentrates rely on differences in size and density to separate milled fractions, and can be expected to have less impact on VOC composition, though heating can still occur as part of the milling process (Mohammad et al. 2016). Finally, differences in VOC composition and off‐flavor expression have also been noted between different commercially available pea protein concentrates and isolates, indicating that specific processing differences or starting materials also contribute toward determining final sensory characteristics (Liu et al. 2023).

Previous research has demonstrated that a wide range of factors and processing strategies can influence the volatile composition and flavor characteristics of pea ingredients. However, many studies have focused primarily on characterizing the VOC composition of pea materials (Murray et al. 1976; Bhowmik et al. 2023; Asavajaru et al. 2025) or used human panels to assess off‐flavors in isolated pea ingredients (El Youssef et al. 2020; Liu et al. 2023). In contrast, fewer studies have investigated how pea ingredients contribute to off‐flavors of more complex food products (Viana and English 2021). Therefore, the goals of this study were (i) to identify the flavor‐related components in a diverse set of value‐added pea ingredients and (ii) to incorporate selected ingredients in a pea‐enriched bread formulation to determine their impact within a conventional food system. These findings aim to inform the selection of relevant parameters to monitor for off‐flavor development in pea materials in future product development work.

## 2 Materials and Methods

### 2.1 Sample Collection

YP seeds (CDC Lewochko variety) were sourced from a farm in Saskatoon, SK, Canada (2022 harvest). The seeds, initially at 6.00% moisture, were subjected to two different thermal treatments, IR, and RF. The IR treatment process utilized a laboratory‐scale IR micronizer (Micronizing Company UK Ltd, Suffolk, UK) with a Model A 156379‐B0 FMC Syntron vibrating conveyor and feeder (Bulk Handling Equipment, Homer City, PA, USA). Seeds were adjusted to a moisture level of 15.12% before heating to 125–130°C, which resulted in a final moisture content of 9.17%. For the RF treatment, a Gentle Processing‐15 lab‐scale food processor (Quantum Mechanical Technology Inc., Prince Albert, SK, Canada) was used. Seeds were adjusted to a moisture level of 13.27% and then heated to 101.3°C, resulting in a final moisture content of 9.99%.

Following thermal treatment, both seed batches were tempered to a moisture content of 12.00%, dehulled using a stone mill (Bühler Stone Mill MJSG), and then milled using a hammer mill (Comminutor, FitzMill, The Fitzpatrick Company, Illinois, USA), followed by fine milling with a roller mill (Bühler Laboratory Mill, Bühler Company, Uzwil, Switzerland). Dehulling and milling was conducted at Cereals Canada (Winnipeg, MB, Canada). Twelve additional YP ingredients were obtained from six suppliers, including three YP protein concentrates (YPC‐1 to YPC‐3) and nine YP protein isolates (YPI‐1 to YPI‐9). A list of all the samples included in this study, as well as their reported protein contents is provided in Table 1. A sample of wheat flour (WF) (ADM Harvest Edge Strong Bakers Untreated Flour 3 STARS) was also sourced from BakeMark (Winnipeg, MB, Canada) and analyzed with the YP samples.

**TABLE 1:** Composition and product type of analyzed yellow pea ingredients.

Sample code	Bread ratio (sample: wheat)	Protein content (%)^a^	Product type
**YP‐IR**	20:80	28.0%	Pea flour
**YP‐RF**	20:80	27.1%	Pea flour
**YPI‐1**	10:90	86%–88%	Pea isolate
**YPI‐2**	10:90	80%–90%	Pea isolate
**YPI‐3**	Not made into bread	88%–90%	Pea isolate
**YPI‐4**	Not made into bread	86%–88%	Pea isolate
**YPI‐5**	Not made into bread	80%	Pea isolate
**YPI‐6**	Not made into bread	>84%	Pea isolate
**YPI‐7**	Not made into bread	90%	Pea isolate
**YPI‐8**	Not made into bread	>90%	Pea isolate
**YPI‐9**	Not made into bread	Not reported	Pea isolate
**YPC‐1**	Not made into bread	>55%	Pea concentrate
**YPC‐2**	Not made into bread	55%–60%	Pea concentrate
**YPC‐3**	Not made into bread	50%	Pea concentrate
**WF**	0:100	13%	Wheat flour

Abbreviations: WF, wheat flour; YPC, yellow pea concentrate; YPI, yellow pea isolate; YP‐IR, yellow pea infrared; YP‐RF, yellow pea radio frequency.

^a^ From product brochures.

### 2.2 YP Ingredient Characterization

#### 2.2.1 Fatty Acids

Analysis of fatty acids in YP ingredients was conducted following the methods described by Fahmi et al. (2021). Lipid contents were extracted using a 2:1 chloroform–methanol (v/v) solution as outlined in Folch et al. (1957), followed by saponification and methylation to produce fatty acid methyl esters. These were then separated on a Varian 450GC gas chromatograph equipped with a flame ionization detector (Varian Canada Inc., Mississauga, ON, Canada), the column temperature was initially set to 100°C and then increased to 175°C at a rate of 25°C/min, where it was held for 30 min. Temperature was then further increased to 220°C at 15°C/min and held for 10 min, followed by a final increase to 240°C at 20°C/min and held for 11 min. The total running time for each sample was 60 min. The GC was operated with a split ratio of 10:1, a column flow rate of 1.8 mL/min, and hydrogen as the carrier gas. Extractions and GC‐analyses were conducted in triplicate for each sample.

#### 2.2.2 Lipoxygenase Activity

Enzyme activity assays for LOX were conducted following the methods described by Bhowmik et al. (2023). The LOX substrate solution was prepared by dissolving 4.5 µL of linoleic acid in 500 µL of 50 mM sodium phosphate buffer (pH 6.8), containing 2% (v/v) Tween‐20 to obtain a 30 mM solution. The crude LOX solution was prepared by suspending 100 mg of YP ingredients in 1 mL of 50 mM sodium phosphate buffer (pH 6.8) containing 4 mM sodium sulfite and 2 mM sodium ascorbate, followed by fractionation. The resulting pellets were then suspended in 500 µL of 50 mM sodium phosphate buffer (pH 6.8). Protein concentration was also determined from the crude LOX fraction by measuring direct absorbance at 280 nm using a NanoDrop One UV‐VIC spectrophotometer (Thermo Fisher, Waltham, USA).

Reaction mixtures consisted of 5 µL of the crude LOX extract, 2 µL of substrate solution, and 200 µL aerated phosphate buffer (pH 6.8). The reaction mixture was vortexed and absorbance at 234 nm measured, followed by a second reading after 5 min. One unit of LOX activity was defined as an increase in absorbance of 0.001 at 234 nm. Results were averaged from triplicate assays and expressed as specific activity per mg protein, with error calculated using appropriate propagation methods.

#### 2.2.3 Volatile Organic Compounds

Extraction of VOCs from the various samples was achieved using solid phase microextraction (SPME) and analyzed with gas chromatography–mass spectrometry (GC–MS) following previously reported protocols (Ryland et al. 2024). A Pyrex bottle was used to mix 5 g of the sample with 90 mL of MilliQ water, 10 g of NaCl, and 200 ng of 1,2‐dichlorobenzene as an internal standard. The resulting mixture was then stirred and heated to 90°C, and VOCs extracted by exposing a 75 µm Carboxen PDMS fiber (SUPELCO, Bellefonte, PA, USA) into the headspace above the mixture for 1 h, before injection into a 7890B gas chromatograph and 7000 GC/Triple Q mass spectrometer (Agilent Technologies, Santa Clara, CA, USA).

Volatile compounds were analyzed using an HP‐5MS column (5%‐phenyl methylpolysiloxane; 30 m × 250 mm × 0.25 mm). Helium (99.999% purity) was used as the carrier gas at an initial flow of 3 mL/min, with nitrogen (99.999% purity) as the make‐up gas. The oven temperature was initially set at 40°C for 5 min, then increased at a rate of 4°C/min to 200°C, held for 1 min, and subsequently increased at 10°C/min to 270°C, where it was maintained for 1 min. The injector was maintained at 250°C and operated in splitless mode with an injection volume of 1 µL. The mass spectrometer was operated in electron‐impact mode at an ionization energy of 70 eV, with the GC–MS transfer line and ion source temperatures set at 250°C and 230°C, respectively. Spectra were collected in full‐scan mode over a mass‐to‐charge (*m*/*z*) range of 29–500.

The peak identities on the resulting spectra were determined by comparison to those of the authentic compounds analyzed and reported in the National Institute of Standards and Technology (NIST version 2.3, 2017) compound library, as well as by matching relative retention indices to those for a set of n‐alkane standards (C8–C20, 40 mg/L in 150 µL of pentane). Results were averaged from quadruplicate runs, and semiquantification of compound concentrations was determined by comparison to the internal standard.

#### 2.2.4 Electronic Nose Responses

Electronic nose (eNose) response data were collected following a previously developed method (Ryland et al. 2024). For measurements, 2.0 g of each sample was placed in a 20 mL borosilicate headspace vial, which was tightly capped and stored at −20°C. Prior to measurement, the vials were allowed to thaw at room temperature for 120 min. Clean air samples, containing no sample material, were prepared alongside the flour samples to serve as controls. Five replicate vials were prepared and analyzed for each sample, except for samples YPI‐6, YPC‐3, YPI‐7, YPI‐8, and YPI‐9, which had two replicates each due to a shortage of sample material. Measurements were performed using an MSEM 160 electronic nose (Sensigent LLC, Baldwin Park, CA, USA) with a headspace sampling apparatus. This setup included a 16 cm‐long, 5 mm‐diameter rubber tube connected to the sampling port of the MSEM 160, and a needle used to pierce the septa caps of the headspace vials. Sampling occurred over 90 s, with an additional 90 s of pre‐ and postsample purging to eliminate carry‐over effects. Five consecutive measurements were taken from each vial without removing the sampling apparatus. To ensure accuracy, clean air cycles were also run between different sample measurements to reset the device and prevent cross‐contamination.

### 2.3 Bread Preparation

To create the bread formulations, the YP‐IR and YP‐RF flours were blended with WF at 20:80 ratios, while YPI‐1 and YPI‐2 samples were included at 10:90 ratios. A control bread was also prepared using 100% WF. A list of all the ingredients used in pan bread formulations is included in Table 2. Breads were produced at a pilot‐scale bakery (Cereals Canada, Winnipeg, MB, Canada) using a no‐time dough baking procedure. The total batch size for each formulation was 3000 g. Ingredients were mixed using a spiral mixer (Erka Model S35, Germany) for 4 min on slow speed, followed by high‐speed mixing until full dough development was achieved. The dough was then rested for 10 min at ambient conditions, scaled into 640 g pieces, and rounded. The dough balls were rested again for 10 min, shaped using a B&B molder (Canadian patent 804301), and fully proofed in a proofer (LBC Bakery Equipment Inc., Model LRP1, Tulalip, WA) at 37°C and 85% relative humidity (RH). The proofed dough samples were baked in a Reel oven (Picard Ovens Inc., Model MT‐8‐24, Drummondville, QC, Canada) at 200°C for 25 min. Once baked, the bread was cooled, sliced into 16 mm using a commercial bread slicer (Oliver Packaging & Equipment Co, Grand Rapids, MI, USA), individually packaged in plastic bags with twist ties, and labeled with loaf numbers and treatment information. The bread samples were stored at room temperature for no longer than 3 h before being transferred to storage at −18°C.

**TABLE 2:** Formulation of pan breads prepared using selected yellow pea ingredients and wheat flour.

Ingredient	Bread formulation (Baker's %)^a^				
	WF	YP‐IR	YP‐RF	YPI‐1	YPI‐2
Flour^b^	100	100	100	100	100
Water	69	78	73	88	87
Sugar, refined^c^	4	4	4	4	4
Shortening^d^	4	4	4	4	4
Salt^e^	1.03	1.03	1.03	1.03	1.03
Yeast, fresh^f^	4	4	4	4	4
Milk powder^g^	2	2	2	2	2
No‐time conditioner^h^	2	2	2	2	2
Gluten^i^	0	9.05	7.05	6	6

Abbreviations: WF, wheat flour; YPC, yellow pea concentrate; YPI, yellow pea isolate; YP‐IR, yellow pea infrared; YP‐RF, yellow pea radio frequency.

^a^ Ingredient weights are expressed as percentages of the flour weight, which is set at 100%.

^b^ As described in Section 2.

^c^ Obtained from Lantic, Montreal, QC, Canada.

^d^ Obtained from Richardson Oilseed, Winnipeg, MB, Canada.

^e^ Obtained from Compass Minerals Canada Corp., Unity, SK, Canada.

^f^ Obtained from AB Mauri (Canada) Limited, Lasalle, QC, Canada.

^g^ Obtained from Lactalis Canada Inc., Laverlochère, QC, Canada.

^h^ Obtained from Puratos Canada Inc., Mississauga ON, Canada.

^i^ Obtained from Dinavedic, Toronto, ON, Canada.

### 2.4 Bread Evaluation

#### 2.4.1 Proximate Analysis

Determination of the proximate composition of the breads was conducted at Central Testing Laboratory Ltd. (Winnipeg, MB, Canada) for percentage of moisture (AOAC 930.15; AOAC 2023a), crude protein (AOAC 990.03; AOAC 2005), crude fiber (AOCS Ba 6a‐05; AOCS 2017b), fat (AOCS AM 5‐04; AOCS 2017a), and ash (AOAC 942.05; AOAC 2023b). Carbohydrate content was estimated as 100 − [(protein) + (fat) + (ash) + (moisture)]. Calories (cal/100 g) were estimated as (4 × [protein]) + (9 × [fat]) + 4 × (100 − [(protein) + (fat) + (ash) + (moisture)] (Fahmi et al. 2019). Triplicate measurements were performed for each sample.

#### 2.4.2 Sensory Analysis

To determine the intensities of the aroma, flavor, texture, and appearance attributes of the bread samples, a trained panel was conducted following the modified Quantitative Descriptive Analysis Method (Stone et al. 2021). The relationship of these attributes to the acceptance of the breads by untrained panelists is critical for further product development work. These two studies were held independently with the consumer study following the descriptive analysis.

##### 2.4.2.1 Sample Preparation and Presentation

The morning on the day of evaluation the bread slices were removed from the freezer 5–10 min before preparation and placed in 26.8 × 27.3 cm Ziploc bags (SC Johnson and Son, Limited, Brantford, ON, Canada). Samples were labeled with three‐digit codes for identification and were otherwise devoid of any other suggestive information. For consumer acceptability, bread slices were cut horizontally about 4 cm from the bottom crust then halved vertically and placed in labeled resealable snack bags (16.5 × 8.2 cm; Loblaws Inc., Toronto, ON, Canada). For descriptive analysis, two 3 × 3 cm pieces were cut from the crumb of the bottom of the bread slice for texture evaluation and placed in capped plastic cups (96 mL; Pactiv Corporation, Forest Lake, IL, USA). For aroma and flavor/taste evaluation, two 3 × 8 cm pieces of crumb were cut from the top half of the slice and placed in snack bags. Appearance samples (4 × 4 cm) were cut from another slice of bread and placed in 96 mL capped plastic cups. Samples were held at room temperature (20°C) for no longer than 3 h before evaluation. All evaluations were performed on the bread crumb.

##### 2.4.2.2 Recruitment

Panelists for consumer (*n* = 65) and descriptive (*n* = 11) panels were recruited through email contact and provided signed consent forms. Eligibility required panelists to be 18 years or older and without food allergies. Recruitment targeted students, staff, and associates at the University of Manitoba's Faculty of Agricultural and Food Sciences. Procedures regarding the recruitment of panelists for consumer acceptability and descriptive analysis panels were approved by the University of Manitoba Human Research Ethics Board 2 (Protocol HS24784‐R2‐2021:027), and an honorarium was provided before the start of the session of the consumer study and the first session of the descriptive analysis study.

##### 2.4.2.3 Consumer Acceptability

Panelists evaluated the sample liking on appearance, aroma, flavor, texture, and overall acceptability using a 9‐point hedonic scale, where 1 = dislike extremely, and 9 = like extremely. The food action rating scale (FACT; Schutz 1965), another rating of acceptability, was also presented, where 1 = I would eat this only if forced, and 9 = I would eat this every opportunity I had. Panelists were asked to respond to questions regarding gender, age, and how often they consume foods containing mostly YPs, either whole or in flour form. Evaluations took place in temperature controlled (20 ± 1°C) individually partitioned workstations equipped with overhead fluorescent lighting and tablets for accessing the questionnaire (Compusense Sensory Software Program; Guelph, ON, Canada). The five breads were presented in balanced order generated by the program. Filtered water was provided for cleansing the palate.

##### 2.4.2.4 Descriptive Analysis

Eleven panelists were recruited to take part in eight 45‐min training sessions and three experimental sessions. Eligibility criteria included no allergies to food or beverages, interest in the study, no knowledge of the study, availability, and at least 18 years of age. No other screening was used for panelist selection. The training sessions were facilitated by an experienced moderator. All samples were coded with different three‐digit random numbers for all sessions. Initially, sensory attributes of the bread samples were recorded by individuals, followed by a discussion of their appropriateness and the technique for evaluation. Aroma attributes were determined first, followed by flavor, taste, texture, and appearance. Their intensities were marked on a 15‐cm line scale with end points labeled low on the left end of the line and high on the right end of the line corresponding to numerical values of 0 and 15 respectively for statistical analysis. Standard samples were presented to illustrate the attributes selected for the final evaluation. Attribute definitions, techniques for evaluating and standard sample details are shown in Table 3. Results shared from repeated sample presentations during the training sessions provided the opportunity to clarify possible reasons for outliers and high variability among panelists and increase the reliability of collected data (Stone et al. 2021). Variability decreased to a degree from the first to second replicate and tended to stay relatively stable from the second to third replicate.

**TABLE 3:** Attribute definitions, methods for evaluation, standard composition, preparation methods, amounts, and manufacturers

Attribute	Definition	Method for Evaluation	Standard Composition	Preparation	Amount in 96 mL Covered Plastic Cup	Manufacturer
*Aroma*		Place the sample bag in position for sniffing. Open the bag. Take three short sniffs. Evaluate the aroma and mark the attribute intensity on the line scale. Close the bag.				
Wheaty	Aroma associated with wheat kernels		Pearled soft wheat	Add 20 g filtered water to 200 g wheat. Rest for 1 hr at room temperature. Process in food processor (Cuisinart Mini‐Prep Plus, Woodbridge ON L4H 0L2) for 2 min	15 g	Trophy Foods, Inc. Mississauga ON
Sweet	Aroma associated with graham wafer		Graham wafer	Cut into 1.5 x 1.5 cm pieces.	3 pieces	HoneyMaid Brand, Christie Brown & Co., Mondelez Canada Inc., Toronto ON
Flour	Aroma associated with whole wheat flour slurry		Whole wheat flour slurry	1 part whole wheat flour thoroughly mixed into 2 parts filtered water	15 g	Western Family Brand produced in Armstrong, BC with 100% Canadian grain; prepared for Save‐On Foods, Vancouver BC
White Bread	Aroma associated with crumb from white bread		White bread	Remove bottom and side crust from crumb and cut into 2 x 2 cm pieces	4 pieces	Wonder Brands Inc., North York ON
Pea	Aroma associated with cooked split yellow pea		Split yellow pea	Combine 1 part dry split yellow pea with 2 parts water. Bring to boil and simmer adding 20% water during the cooking process. Cook until tender about 30 to 35 min. Cool slightly, add 10% water and puree in food processor (Cuisinart, Mini‐Prep Plus, 100 Conair Parkway, Woodbridge, ON)	15 g	Quality Natural Foods Canada Inc., Scarborough ON
*Flavor/Taste*		Flavor ‐ Take a bite of the sample. Chew the sample thoroughly ensuring that the sample reaches all surfaces of your mouth. Evaluate the flavor/taste and mark the attribute intensity on the line scale just before swallowing the sample. Taste ‐ Take a sip of the sample ensuring that the sample reaches all of the surfaces of your mouth.				
Wheaty	Flavor associated with the aroma of wheat kernels		As above for aroma			
White Bread	Flavor associated with white bread crumb		As above for aroma			
Milky	Flavor associated with milk		Milk 3% fat	Equilibrate to room temperature (∼20C)	20 g	Dairyland Brand, Saputo Inc., Montreal QC
Pea	Flavor associated with cooked split yellow pea		As above for aroma			
Sweet Taste	Taste associated with sucrose in aqueous solution		1% sucrose solution	25 g white granulated sugar dissolved in 250 mL filtered water	15 g (amt in 29 mL covered plastic cup)	Rogers Brand, Lantic Inc., Montreal QC
Sour Taste	Taste associated with citric acid in aqueous solution		0.02% citric acid solution	0.1 g citric acid dissolved in 500 mL filtered water	15 g (amt in 29 mL covered plastic cup)	Teja Brand imported by Bains Wholesale Foods Ltd., Surrey BC
*Texture*						
Springiness	Force with which the sample returns to its original size/shape after partial compression (without failure) between the molars.	Place the sample between the molars and press lightly on the surface to partially compress the sample and then raise molars.	White bread	Remove bottom and side crust from crumb and cut into 3 x 3 cm pieces	3 pieces	Wonder Brand Inc., North York ON
Firmness	The amount of force to compress the sample fully.	Place the sample between the molars and compress the sample.	Rye bread	Remove bottom and side crust from crumb and cut into 3 x 3 cm pieces	3 pieces	City Bread Co. Ltd., Winnipeg MB
Denseness	Compactness of the cross section of the sample after biting completely through with the molars.	Place the sample between the molars and bite through the sample.	Plain bagel	2 cm piece from top half of pre‐cut piece	3 pieces	Dempster's Original Bagel, Bimbo Canada, Etobicoke ON
Chewiness	The length of time required to masticate the sample at a constant rate of force application to reduce it to a consistency suitable for swallowing.	Place sample in mouth and masticate at 1 chew per second. Determine the degree of chewiness as the number of chews required before the product is ready for swallowing.	Unsalted top saltine	2.5 × 2.5 cm (1/4 cracker)	3 pieces	Premium Plus Brand, Christie Brown & Co., Mondelez Canada Inc., Toronto ON
Adhesiveness to Teeth	Amount of product adhering on (top) or in (crevices) the molars after swallowing the sample.	Chewing is done on the same side of the mouth and evaluation is made after swallowing the sample.	Graham wafer	1.5 × 1.5 cm (1/8 cracker)	3 pieces	HoneyMaid Brand, Christie Brown & Co., Mondelez Canada Inc., Toronto ON
*Appearance*						
Whitish Yellow Color	The intensity of the whitish yellow color of the bread crumb.	Look at the crumb surface that is facing up and mark at the point that corresponds to the intensity of the whitish yellow color where low is whitish and high is yellow within the context of bakery products.	n/a	n/a	n/a	n/a

Following the initial training sessions, it was possible to conduct the experimental sessions on three different days within a 1‐week period as the sample remained stable and the panelists were available. In each experimental session, panelists were presented with the five samples in a random order. Sessions for aroma, flavor/taste, and texture were conducted in individual workstations. To mask possible color differences in the breads that could lead to bias, samples were placed under incandescent light shielded by red plastic film. For appearance evaluation, panelists were instructed to remove the bread square from the portion cup and place it on the outlined square in the center of the 28 × 43 cm piece of white paper. The paper was placed on a light gray table with white foam board partitions (7 mm thick, 61 cm high, 76 cm across the front, 46 cm for the side) between stations. Fluorescent overhead lighting illuminated the room. Filtered water was available throughout both the training and experimental sessions for cleansing the palate.

#### 2.4.3 Instrumental Color

Analysis of color by instrument was performed with a calibrated Lovibond Spectropolarimeter LC100 (The Tintometer Ltd. Lovibond House, Solar Way Solstice Park, Amesbury, UK) using the 8 mm setting. Three bread slices for each sample were thawed at room temperature prior to reading, and three readings for L* (lightness), a* (green–red color), and b* (blue–yellow color) were taken at D65°/10 from random positions within the crumb portion on the surface of each slice.

#### 2.4.4 pH

The pH of the YP ingredients used to make bread was determined using an Orion Star A211 pH meter (Thermo Fisher, Waltham, USA), following a modified version of the AACC method (02‐52.01) due to limited sample availability (AACC 2009). Specifically, 2.0 g of each sample was dispersed into 20 mL of Milli‐Q water and stirred until homogenous using a magnetic stirrer. The calibrated pH probe was then inserted, and stable readings accurate to two decimal places were recorded after holding for 1 min. The pH meter was recalibrated between each new sample, and results were averaged from sextuplicate measurements.

### 2.5 Statistical Analysis

One‐way analysis of variance (ANOVA) was used to assess statistical significance for volatile compounds, fatty acids, LOX activity, color, and proximate composition using SPSS (Version 28, IBM Corp.) and SAS (Version 9.4, SAS Institute Inc.). The significance threshold for these analyses was set at *p* ≤ 0.05, and post hoc comparisons were conducted using Tukey's HSD test. Two‐way and three‐way ANOVA were applied to the data for consumer acceptability and descriptive analysis, respectively, using SAS (Version 9.4, SAS Institute Inc.). For the consumer acceptability data, “consumer” was treated as a random factor, while “sample” was treated as a fixed factor, degree of liking was the dependent variable. For descriptive analysis, random factors included panelist and replication, with sample as a fixed effect, attribute intensity was the dependent variable. Interactions between panelist and replication, panelist and sample, and sample and replication were included unless terms were not significant, in which case they were pooled back into the error term as recommended by O'Mahony (1986). Post hoc multiple comparisons were made using Tukey's HSD test.

To explore relationships among the YP ingredients and derived products, partial least squares regression (PLS‐R) analyses were conducted using XLSTAT (Addinsoft, Paris, France). PLS‐R is an efficient multivariate method optimized for covariance‐based criteria, suitable when the number of explanatory variables is high, and potential correlations exist among them. Two components were retained in each model, and validation was performed using the Jackknife leave‐one‐out method. All variables were centered and standardized prior to analysis and mean values that showed significant differences between samples were included. Biplots were generated to visualize relationships among variables and sample distributions.

#### 2.5.1 YP Ingredient Characterization

The first model examined the relationship between LOX activity (Y variable) and VOCs, fatty acids, and eNose responses (X variables) across the fourteen YP ingredients. The resulting data matrix included 14 rows and one Y column, and 14 rows and 35 X columns. Model quality parameters for cumulative Q^2^, R^2^X, and R^2^Y were 0.545, 0.795, and 0.520, respectively. Variables with variable importance in projection (VIP) scores greater than one—indicating high influence included 1‐hexanol, carvone, octanoic acid ethyl ester, and decanal in both components.

#### 2.5.2 Bread Evaluation

The second model assessed relationships between acceptance of sensory attributes (Y variables) and descriptive attributes, color, proximate composition of the breads, and the fatty acid and VOC profiles of the YP ingredients (X variables). The data matrix contained five rows and four Y columns, and five rows and 58 X columns. Model quality values for cumulative Q^2^, R^2^X, and R^2^Y were 0.853, 0.791, and 0.643, respectively. VIP scores greater than one were obtained for 25 of the 58 X variables, including white bread, pea, and sweet aromas; sweet, milky, white bread, and pea flavors; crude protein, carbohydrate, LOX activity, octanal, nonanal, 1‐hexanol, color, octanoic acid ethyl ester, linolenic acid, furan‐2‐pentyl, 2‐decanone, acetophenone, 2‐undecanone, heptanal, and carvone across both components.

Stepwise linear regression (SPSS Version 28, IBM Corp.) identified the sensory attributes most strongly associated with overall consumer acceptability scores. eNose data were analyzed using CDAnalysis software (Sensigent Intelligent Sensing Systems, Baldwin Park, CA, USA). eNose sensor data were processed using DR/R transformation with area normalization applied. No baseline correction or additional filtering was performed, as preliminary tests showed that these steps did not improve model discrimination. All 32 sensor signals were included in the analysis, and the resulting dataset was mean‐centered before multivariate modeling. For model cross‐validation, canonical discriminant analysis (CDA) was used as the classification algorithm and the optimal PCA count was used. The experimental workflow, outlining the sequence of analyses and evaluations performed, is illustrated in Table 4.

**TABLE 4:** Summary of the workflow used to investigate the flavor and functional properties of diverse yellow pea ingredients.

Stage		Description
I.	Sample collection	Assorted yellow pea (YP) products were obtained: two heat‐treated flours (YP‐RF, YP‐IR), nine YP protein isolates (YPIs), and three YP protein concentrates (YPCs).
II.	YP ingredient characterization	**Flavoromics profiling**: fatty acid composition, lipoxygenase (LOX) activity, volatile organic compounds (VOCs), and electronic nose (eNose) responses were measured. **Statistical analyses**: one‐way ANOVA with Tukey's HSD for fatty acids (Table 5), LOX (Table 5), and VOCs (Table 6); PCA for eNose data (Figure 1A,B); PLS‐R using LOX activity (Y) and selected fatty acids and significant VOCs (X) (Figure 2). **Outcome**: four ingredients (YPI‐1, YPI‐2, YP‐RF, YP‐IR) were selected for bread formulation.
III.	Bread preparation	Breads were prepared using selected YP ingredients: YP‐RF and YP‐IR at 20:80 ingredient:WF; YPI‐1 and YPI‐2 at 10:90 ingredient:WF.
IV.	Bread evaluation	**Sensory analysis**: consumer acceptability (*n* = 65) and descriptive analysis (*n* = 11; eight training, three test sessions). **Instrumental and compositional analyses**: proximate composition, color, and pH. **Statistical analyses**: one‐way ANOVA for proximate composition, pH, and color (Table 7); two‐way ANOVA for consumer acceptability (Table 8); three‐way ANOVA for descriptive data (Table 9); PLS‐R using significant acceptability scores (Y) and significant descriptive, color, proximate, fatty acid, and VOC variables (X) (Figure 3).

Abbreviations: WF, wheat flour; YPC, yellow pea concentrate; YPI, yellow pea isolate; YP‐IR, yellow pea infrared; YP‐RF, yellow pea radio frequency.

## 3 Results and Discussion

### 3.1 YP Ingredient Characterization

#### 3.1.1 Fatty Acids

Fatty acid composition of the various YP ingredients is shown in Table 5. Overall, the fatty acid profiles observed in YP ingredients were similar to one another and consistent with previously reported values (Bhowmik et al. 2023), though significant differences were found between at least two YP ingredients in this study for most of the fatty acids analyzed, and some trends were apparent. For instance, YPI samples tended to display greater variability compared to YPCs and the two treated flours. As an example, oleic acid, the second most abundant fatty acid in the YP samples, varied significantly between different YPIs (20.3%–29.2%), but showed no significant differences between YPCs (24.6%–25.9%) or the two treated flour samples (23.9%–24.1%). Linoleic acid, which dominated the fatty acid contents of the YP samples, similarly showed significant differences between YPIs (42.9%–50.7%), but not YPCs or treated flours. As linoleic acid is a substrate for LOX enzymes, these variations could reflect different degrees of LOX activity a sample has experienced over its lifetime (Bhowmik et al. 2023). In contrast, the lack of significant differences for α‐linolenic acid, another LOX substrate, may suggest the opposite. One explanation for the greater variation in fatty acid contents among YPI samples could be that these ingredients are typically subjected to processing steps intended to modify or reduce fat contents (Shanthakumar et al. 2022). Alternatively, the differences may simply reflect variation in the initial fatty acid composition of source materials or the greater number of YPI samples analyzed (*n* = 9) compared to YPCs (*n* = 3) or treated flours (*n* = 2). In terms of general nutritional characteristics, all YP ingredients differed from WF, exhibiting significantly higher total monounsaturated fatty acid (MUFA) contents, lower polyunsaturated fatty acid (PUFA) contents, and lower ω‐6/ω‐3 fatty‐acid ratio. These differences suggest that even partial replacement of WF with YP ingredients in bread formulations could substantially shift the overall fatty‐acid profile of the product and potentially offer nutritional advantages.

**TABLE 5:** Fatty acid composition and LOX activity of yellow pea ingredients and wheat flour.

Fatty acid (% of total fatty acids)	Source of variation (*F* value^a^, 14, 45 df)^b^	Sample mean values (*n* = 4)^c^					
Lauric acid (C12:0)	18.94 ***	WF	0.00^a^ (0.00)	YPI‐3	0.01^de^ (0.00)	YPI‐8	0.01^ab^ (0.00)
		YP‐IR	0.01^ab^ (0.00)	YPI‐4	0.01^e^ (0.00)	YPI‐9	0.01^ab^ (0.00)
		YP‐RF	0.01^ab^ (0.00)	YPI‐5	0.01^ab^ (0.00)	YPC‐1	0.00^a^ (0.00)
		YPI‐1	0.01^bcd^ (0.00)	YPI‐6	0.01^cde^ (0.00)	YPC‐2	0.01^ab^ (0.00)
		YPI‐2	0.01^bc^ (0.00)	YPI‐7	0.01^ab^ (0.00)	YPC‐3	0.01^bcd^ (0.00)
Myristic acid (C14:0)	118.72 ***	WF	0.10^a^ (0.00)	YPI‐3	0.19^bc^ (0.00)	YPI‐8	0.32^f^ (0.00)
		YP‐IR	0.21^c^ (0.01)	YPI‐4	0.18^bc^ (0.00)	YPI‐9	0.26^e^ (0.00)
		YP‐RF	0.21^cd^ (0.00)	YPI‐5	0.19^bc^ (0.00)	YPC‐1	0.17^b^ (0.00)
		YPI‐1	0.17^b^ (0.00)	YPI‐6	0.24^de^ (0.00)	YPC‐2	0.22^cd^ (0.00)
		YPI‐2	0.22^de^ (0.00)	YPI‐7	0.39^g^ (0.01)	YPC‐3	0.29^f^ (0.04)
Pentadecanoic acid (C15:0)	59.08 ***	WF	0.09^a^ (0.00)	YPI‐3	0.19^d^ (0.00)	YPI‐8	0.19^d^ (0.00)
		YP‐IR	0.16^b^ (0.00)	YPI‐4	0.21^e^ (0.00)	YPI‐9	0.16^b^ (0.00)
		YP‐RF	0.16^b^ (0.00)	YPI‐5	0.16^b^ (0.00)	YPC‐1	0.15^b^ (0.00)
		YPI‐1	0.20^de^ (0.00)	YPI‐6	0.16^bc^ (0.00)	YPC‐2	0.16^b^ (0.00)
		YPI‐2	0.19^de^ (0.00)	YPI‐7	0.19^d^ (0.00)	YPC‐3	0.18^cd^ (0.02)
Palmitic acid (C16:0)	25.97 ***	WF	18.07^g^ (0.36)	YPI‐3	12.06^ab^ (0.06)	YPI‐8	14.37^def^ (0.05)
		YP‐IR	13.47^abcde^ (0.11)	YPI‐4	12.71^abcd^ (0.05)	YPI‐9	15.05^ef^ (0.05)
		YP‐RF	13.59^bcde^ (0.06)	YPI‐5	12.73^abcd^ (0.11)	YPC‐1	11.85^a^ (0.1)
		YPI‐1	12.60^abc^ (0.04)	YPI‐6	13.28^abcd^ (0.16)	YPC‐2	12.66^abc^ (0.49)
		YPI‐2	14.20^bcdef^ (0.13)	YPI‐7	15.68^f^ (0.1)	YPC‐3	15.42^f^ (2.08)
trans‐Palmitoleic acid (C16:1t)	21.28 ***	WF	0.09^b^ (0.01)	YPI‐3	0.03^a^ (0.00)	YPI‐8	0.04^a^ (0.00)
		YP‐IR	0.04^a^ (0.02)	YPI‐4	0.04^a^ (0.00)	YPI‐9	0.03^a^ (0.00)
		YP‐RF	0.03^a^ (0.00)	YPI‐5	0.03^a^ (0.00)	YPC‐1	0.02^a^ (0.00)
		YPI‐1	0.03^a^ (0.00)	YPI‐6	0.04^a^ (0.00)	YPC‐2	0.03^a^ (0.00)
		YPI‐2	0.03^a^ (0.01)	YPI‐7	0.04^a^ (0.00)	YPC‐3	0.04^a^ (0.00)
Palmitoleic acid (C16:1)	40.30 ***	WF	0.09^g^ (0.00)	YPI‐3	0.06^bc^ (0.00)	YPI‐8	0.08^fg^ (0.00)
		YP‐IR	0.05^abc^ (0.00)	YPI‐4	0.06^cd^ (0.00)	YPI‐9	0.04^a^ (0.00)
		YP‐RF	0.05^abc^ (0.00)	YPI‐5	0.06^cd^ (0.00)	YPC‐1	0.05^ab^ (0.00)
		YPI‐1	0.05^abc^ (0.00)	YPI‐6	0.08^g^ (0.00)	YPC‐2	0.07^ef^ (0.00)
		YPI‐2	0.06^cde^ (0.01)	YPI‐7	0.08^fg^ (0.00)	YPC‐3	0.07^def^ (0.01)
Heptadecanoic acid (C17:0)	33.45 ***	WF	0.11^a^ (0.00)	YPI‐3	0.19^bc^ (0.00)	YPI‐8	0.20^bcd^ (0.00)
		YP‐IR	0.20^bcd^ (0.00)	YPI‐4	0.20^bcd^ (0.00)	YPI‐9	0.23^e^ (0.00)
		YP‐RF	0.21^bcde^ (0.00)	YPI‐5	0.21^bcde^ (0.00)	YPC‐1	0.19^b^ (0.00)
		YPI‐1	0.20^bcd^ (0.00)	YPI‐6	0.20^bcd^ (0.00)	YPC‐2	0.19^bc^ (0.01)
		YPI‐2	0.22^cde^ (0.00)	YPI‐7	0.20^bcd^ (0.00)	YPC‐3	0.22^de^ (0.03)
Stearic acid (C18:0)	73.23 ***	WF	1.18^a^ (0.03)	YPI‐3	3.79^bcd^ (0.01)	YPI‐8	4.01^cd^ (0.00)
		YP‐IR	3.34^b^ (0.02)	YPI‐4	3.92^cd^ (0.04)	YPI‐9	4.78^f^ (0.00)
		YP‐RF	3.37^b^ (0.05)	YPI‐5	4.24^de^ (0.01)	YPC‐1	3.73^bc^ (0.01)
		YPI‐1	3.84^bcd^ (0.00)	YPI‐6	4.02^cd^ (0.02)	YPC‐2	4.17^cd^ (0.09)
		YPI‐2	3.95^cd^ (0.01)	YPI‐7	3.88^cd^ (0.02)	YPC‐3	4.72^ef^ (0.64)
Oleic acid (C18:1)	216.53 ***	WF	12.36^a^ (0.25)	YPI‐3	25.35^efg^ (0.04)	YPI‐8	22.06^c^ (0.03)
		YP‐IR	23.93^d^ (0.17)	YPI‐4	25.47^efg^ (0.04)	YPI‐9	20.31^b^ (0.03)
		YP‐RF	24.09^de^ (0.04)	YPI‐5	24.50^bc^ (0.06)	YPC‐1	25.92^g^ (0.13)
		YPI‐1	24.43^def^ (0.02)	YPI‐6	29.20^i^ (0.17)	YPC‐2	24.65^defg^ (0.41)
		YPI‐2	27.75^h^ (0.03)	YPI‐7	20.52^b^ (0.12)	YPC‐3	25.61^fg^ (2.35)
cis‐Vaccenic acid (C18:1n7c)	7.04 ***	WF	0.71^bc^ (0.01)	YPI‐3	0.50^ab^ (0.01)	YPI‐8	0.60^ab^ (0.00)
		YP‐IR	0.46^a^ (0.00)	YPI‐4	0.51^ab^ (0.01)	YPI‐9	0.48^a^ (0.00)
		YP‐RF	0.46^a^ (0.01)	YPI‐5	0.49^ab^ (0.01)	YPC‐1	0.49^ab^ (0.01)
		YPI‐1	0.49^ab^ (0.00)	YPI‐6	0.83^c^ (0.01)	YPC‐2	0.56^ab^ (0.01)
		YPI‐2	0.60^ab^ (0.00)	YPI‐7	0.60^ab^ (0.01)	YPC‐3	0.39^a^ (0.28)
Linoleic acid (C18:2)	6.99 ***	WF	58.13^d^ (0.17)	YPI‐3	47.17^abc^ (0.06)	YPI‐8	49.46^abc^ (0.04)
		YP‐IR	45.38^abc^ (0.34)	YPI‐4	46.46^abc^ (0.09)	YPI‐9	50.71^bc^ (0.02)
		YP‐RF	45.43^abc^ (0.19)	YPI‐5	47.4^abc^ (0.01)	YPC‐1	46.15^abc^ (0.1)
		YPI‐1	47.58^abc^ (0.05)	YPI‐6	42.87^a^ (0.05)	YPC‐2	46.65^abc^ (0.47)
		YPI‐2	43.96^ab^ (0.05)	YPI‐7	49.38^abc^ (0.12)	YPC‐3	52.27^cd^ (9.5)
α‐Linolenic acid (C18:3n3)	8.07 ***	WF	3.15^a^ (0.31)	YPI‐3	8.31^b^ (0.13)	YPI‐8	6.76^b^ (0.01)
		YP‐IR	8.20^b^ (0.11)	YPI‐4	7.97^b^ (0.04)	YPI‐9	6.17^b^ (0.01)
		YP‐RF	8.13^b^ (0.07)	YPI‐5	7.81^b^ (0.04)	YPC‐1	8.44^b^ (0.08)
		YPI‐1	8.31^b^ (0.02)	YPI‐6	5.96^b^ (0.03)	YPC‐2	7.58^b^ (0.14)
		YPI‐2	6.02^b^ (0.07)	YPI‐7	6.89^b^ (0.01)	YPC‐3	6.41^b^ (3.32)
Arachidic acid (C20:0)	8.31 ***	WF	0.16^a^ (0.06)	YPI‐3	0.48^bc^ (0.01)	YPI‐8	0.51^bc^ (0.00)
		YP‐IR	0.58^c^ (0.2)	YPI‐4	0.47^bc^ (0.00)	YPI‐9	0.43^bc^ (0.00)
		YP‐RF	0.47^bc^ (0.00)	YPI‐5	0.44^bc^ (0.01)	YPC‐1	0.39^b^ (0.02)
		YPI‐1	0.44^bc^ (0.00)	YPI‐6	0.51^bc^ (0.00)	YPC‐2	0.46^bc^ (0.01)
		YPI‐2	0.44^bc^ (0.01)	YPI‐7	0.55^bc^ (0.00)	YPC‐3	0.55^bc^ (0.08)
Gondoic acid (C20:1)	12.16 ***	WF	0.44^c^ (0.02)	YPI‐3	0.36^bc^ (0.00)	YPI‐8	0.33^ab^ (0.00)
		YP‐IR	0.29^ab^ (0.00)	YPI‐4	0.37^bc^ (0.00)	YPI‐9	0.31^ab^ (0.00)
		YP‐RF	0.29^ab^ (0.00)	YPI‐5	0.29^ab^ (0.00)	YPC‐1	0.27^a^ (0.01)
		YPI‐1	0.34^ab^ (0.00)	YPI‐6	0.43^c^ (0.01)	YPC‐2	0.30^ab^ (0.00)
		YPI‐2	0.37^bc^ (0.02)	YPI‐7	0.33^ab^ (0.01)	YPC‐3	0.44^c^ (0.10)
Eicosadienoic acid (C20:2)	11.04 ***	WF	0.06^f^ (0.00)	YPI‐3	0.06^ef^ (0.00)	YPI‐8	0.05^abc^ (0.00)
		YP‐IR	0.06^cdef^ (0.00)	YPI‐4	0.06^def^ (0.00)	YPI‐9	0.05^bcde^ (0.00)
		YP‐RF	0.05^bcde^ (0.00)	YPI‐5	0.05^abc^ (0.00)	YPC‐1	0.05^abcd^ (0.00)
		YPI‐1	0.06^def^ (0.00)	YPI‐6	0.04^ab^ (0.00)	YPC‐2	0.05^bcd^ (0.00)
		YPI‐2	0.04^a^ (0.01)	YPI‐7	0.05^abc^ (0.00)	YPC‐3	0.06^cdef^ (0.01)
Eicosapentaenoic acid (C20:5)	31.71 ***	WF	0.08^ab^ (0.01)	YPI‐3	0.05^abc^ (0.01)	YPI‐8	0.09^abc^ (0.00)
		YP‐IR	0.04^ab^ (0.00)	YPI‐4	0.09^abc^ (0.00)	YPI‐9	0.16^bc^ (0.01)
		YP‐RF	0.04^ab^ (0.01)	YPI‐5	0.11^abc^ (0.01)	YPC‐1	0.03^a^ (0.01)
		YPI‐1	0.10^abc^ (0.01)	YPI‐6	0.42^d^ (0.03)	YPC‐2	0.09^abc^ (0.01)
		YPI‐2	0.48^d^ (0.03)	YPI‐7	0.13^abc^ (0.02)	YPC‐3	0.17^c^ (0.15)
Docosanoic acid (C22:0)	34.40 ***	WF	0.21^d^ (0.01)	YPI‐3	0.14^ab^ (0.00)	YPI‐8	0.17^c^ (0.00)
		YP‐IR	0.13^ab^ (0.00)	YPI‐4	0.14^ab^ (0.01)	YPI‐9	0.14^b^ (0.00)
		YP‐RF	0.13^ab^ (0.00)	YPI‐5	0.13^ab^ (0.00)	YPC‐1	0.12^a^ (0.00)
		YPI‐1	0.13^ab^ (0.01)	YPI‐6	0.17^c^ (0.00)	YPC‐2	0.14^ab^ (0.01)
		YPI‐2	0.18^c^ (0.01)	YPI‐7	0.19^cd^ (0.00)	YPC‐3	0.18^c^ (0.02)
Erucic acid (C22:1)	36.05 ***	WF	0.05^de^ (0.01)	YPI‐3	0.06^efg^ (0.00)	YPI‐8	0.06^efg^ (0.00)
		YP‐IR	0.06^def^ (0.00)	YPI‐4	0.06^efg^ (0.00)	YPI‐9	0.04^bc^ (0.00)
		YP‐RF	0.05^cd^ (0.00)	YPI‐5	0.03^ab^ (0.00)	YPC‐1	0.02^a^ (0.00)
		YPI‐1	0.05^de^ (0.01)	YPI‐6	0.03^ab^ (0.00)	YPC‐2	0.05^de^ (0.01)
		YPI‐2	0.04^cd^ (0.00)	YPI‐7	0.07^fg^ (0.00)	YPC‐3	0.07^g^ (0.01)
Lignoceric acid (C24:0)	2.60 *	WF	0.23^abc^ (0.01)	YPI‐3	0.24^abc^ (0.01)	YPI‐8	0.32^bc^ (0.01)
		YP‐IR	0.17^a^ (0.01)	YPI‐4	0.24^abc^ (0.00)	YPI‐9	0.34^c^ (0.00)
		YP‐RF	0.17^a^ (0.00)	YPI‐5	0.28^abc^ (0.01)	YPC‐1	0.26^abc^ (0.01)
		YPI‐1	0.22^ab^ (0.00)	YPI‐6	0.30^bc^ (0.00)	YPC‐2	0.33^bc^ (0.01)
		YPI‐2	0.29^bc^ (0.00)	YPI‐7	0.33^bc^ (0.01)	YPC‐3	0.29^bc^ (0.23)
Nervonic acid (C24:1)	11.55 ***	WF	0.06^d^ (0.00)	YPI‐3	0.03^bc^ (0.00)	YPI‐8	0.03^abc^ (0.00)
		YP‐IR	0.02^ab^ (0.00)	YPI‐4	0.03^bc^ (0.00)	YPI‐9	0.02^abc^ (0.00)
		YP‐RF	0.02^ab^ (0.00)	YPI‐5	0.02^abc^ (0.00)	YPC‐1	0.01^a^ (0.00)
		YPI‐1	0.03^abc^ (0.00)	YPI‐6	0.03^bc^ (0.00)	YPC‐2	0.02^ab^ (0.00)
		YPI‐2	0.03^bc^ (0.00)	YPI‐7	0.04^c^ (0.02)	YPC‐3	0.03^abc^ (0.00)
SFA	31.46 ***	WF	20.14^bc^ (0.45)	YPI‐3	17.29^a^ (0.08)	YPI‐8	20.09^bc^ (0.07)
		YP‐IR	18.26^ab^ (0.09)	YPI‐4	18.08^ab^ (0.11)	YPI‐9	21.39^c^ (0.06)
		YP‐RF	18.31^ab^ (0.1)	YPI‐5	18.38^ab^ (0.11)	YPC‐1	16.85^c^ (0.08)
		YPI‐1	17.80^ab^ (0.04)	YPI‐6	18.89^abc^ (0.19)	YPC‐2	18.34^ab^ (0.56)
		YPI‐2	19.70^bc^ (0.14)	YPI‐7	21.34^c^ (0.01)	YPC‐3	20.25^bc^ (2.01)
MUFA	177.48 ***	WF	13.80^a^ (0.3)	YPI‐3	26.40^d^ (0.05)	YPI‐8	23.19^c^ (0.02)
		YP‐IR	24.83^d^ (0.16)	YPI‐4	26.54^d^ (0.05)	YPI‐9	21.23^b^ (0.03)
		YP‐RF	24.98^d^ (0.04)	YPI‐5	25.43^d^ (0.06)	YPC‐1	26.79^d^ (0.14)
		YPI‐1	25.43^d^ (0.02)	YPI‐6	30.65^f^ (0.17)	YPC‐2	25.70^d^ (0.42)
		YPI‐2	28.88^e^ (0.05)	YPI‐7	21.65^b^ (0.14)	YPC‐3	26.77 (2.57)
PUFA	34.41 ***	WF	61.42^d^ (0.46)	YPI‐3	55.60^bc^ (0.09)	YPI‐8	56.37^c^ (0.05)
		YP‐IR	53.68^abc^ (0.28)	YPI‐4	54.58^abc^ (0.13)	YPI‐9	57.09^c^ (0.03)
		YP‐RF	53.66^abc^ (0.27)	YPI‐5	55.36^bc^ (0.04)	YPC‐1	54.67^abc^ (0.18)
		YPI‐1	56.05^bc^ (0.07)	YPI‐6	49.29^a^ (0.05)	YPC‐2	54.36^abc^ (0.42)
		YPI‐2	50.49^a^ (0.14)	YPI‐7	56.46^c^ (0.15)	YPC‐3	52.01^ab^ (4.39)
PUFA/SFA	17.18 ***	WF	3.05^bcd^ (0.09)	YPI‐3	3.21^cd^ (0.02)	YPI‐8	2.81^abcd^ (0.01)
		YP‐IR	2.94^abcd^ (0.03)	YPI‐4	3.02^bcd^ (0.02)	YPI‐9	2.67^abc^ (0.01)
		YP‐RF	2.93^abcd^ (0.03)	YPI‐5	3.01^bcd^ (0.02)	YPC‐1	3.25^d^ (0.02)
		YPI‐1	3.15^cd^ (0.01)	YPI‐6	2.61^ab^ (0.03)	YPC‐2	2.97^bcd^ (0.08)
		YPI‐2	2.56^a^ (0.02)	YPI‐7	2.65^abc^ (0.01)	YPC‐3	2.59^ab^ (0.47)
Omega 6/Omega 3 ratio	125.69 ***	WF	18.60^b^ (1.9)	YPI‐3	5.69^a^ (0.1)	YPI‐8	7.33^a^ (0.01)
		YP‐IR	5.54^a^ (0.11)	YPI‐4	5.84^a^ (0.02)	YPI‐9	8.25^a^ (0.01)
		YP‐RF	5.59^a^ (0.03)	YPI‐5	6.08^a^ (0.03)	YPC‐1	5.47^a^ (0.04)
		YPI‐1	5.74^a^ (0.01)	YPI‐6	7.27^a^ (0.03)	YPC‐2	6.17^a^ (0.15)
		YPI‐2	7.39^a^ (0.07)	YPI‐7	7.18^a^ (0.02)	YPC‐3	6.79^a^ (0.03)
LOX activity (unit activity/mg protein)	8.51 ***	WF	1.07^a^ (0.04)	YPI‐3	2.11^ab^ (0.21)	YPI‐8	1.81^ab^ (0.56)
		YP‐IR	1.31^a^ (0.54)	YPI‐4	2.21^ab^ (0.96)	YPI‐9	1.9^ab^ (0.23)
		YP‐RF	1.54^a^ (0.13)	YPI‐5	1.31^a^ (0.07)	YPC‐1	5.88^c^ (1.67)
		YPI‐1	2.15^ab^ (0.51)	YPI‐6	1.53^a^ (0.22)	YPC‐2	2.84^ab^ (0.48)
		YPI‐2	2.33^ab^ (0.5)	YPI‐7	1.49^a^ (0.37)	YPC‐3	3.77^bc^ (1.59)

Abbreviations: WF, wheat flour; YPC, yellow pea concentrate; YPI, yellow pea isolate; YP‐IR, yellow pea infrared; YP‐RF, yellow pea radio frequency.

^a^ NS, not significant; *p* ≥ 0.05; ∗*p* < 0.05, ∗∗∗*p* < 0.001, mean values (followed in brackets by the standard deviation) within the fatty acid or LOX activity with the same letter are not significantly different when a probability level of 0.05 is applied.

^b^ Except for LOX activity where *F* value was 14, 30 df.

^c^ Except for LOX activity where mean values *n* = 3.

#### 3.1.2 Lipoxygenase Activity

LOX activity has been identified with the formation of “off‐flavors” in pulses such as YP, and therefore, reducing or eliminating the activity of these enzymes is expected to provide sensory benefits for products made using such ingredients (Roland et al. 2017; Bhowmik et al. 2023). Among the 14 YP samples analyzed, 12 exhibited LOX activities ranging from 1.31 to 2.84 U/mg protein, comparable to that of the WF sample (1.07 U/mg protein; Table 5). However, two YPCs, YPC‐1 and YPC‐3, showed significantly higher activities at 5.88 and 3.77 U/mg protein, respectively.

Despite this, all YP samples analyzed demonstrated a 20‐ to 100‐fold reduction in LOX activity compared to unprocessed wild‐type YP flour (170.18 U/mg protein) in a previous study (Bhowmik et al. 2023), indicating that the processing methods used to produce these ingredients were highly effective at suppressing enzyme activity. The lack of significant activity differences between YPIs was somewhat unexpected, as a previous study observed that a 0.5 pH difference at one stage of isolate production significantly altered final LOX activity, suggesting that small differences in production methods could conceivably produce highly variable LOX activities (Gao et al. 2020). In contrast, two of the three YPCs displayed significantly higher enzyme activity levels, implying that the processing conditions used to create these ingredients were more variable in their destructive effects toward LOX enzymes. However, the LOX activity reported in this study reflects only the remaining enzyme activity in the ingredients at the time of testing, and it does not account for the cumulative activity the enzyme may have exerted earlier in the product's lifetime.

#### 3.1.3 Volatile Organic Compounds

In total, 35 individual VOCs were detected by GC–MS analysis in each of the 14 YP samples and WF, among which 31 were definitively identified. A full list of these VOCs, their relative concentrations (µg/100 g), and their known odor descriptions is reported in Table 6; the retention time, LRIs, and ions used to assess the concentration for these compounds are presented in Supporting Information Table S1. Many of these VOCs have been previously identified as important aroma‐active VOCs, or potential off‐flavor contributors in pea‐derived materials, including hexanal, heptanal, 1‐octen‐3‐ol and octanal (Bhowmik et al. 2023; Fahmi et al. 2021; Liu et al. 2023); 2‐hexenal, furan‐2‐pentyl, and (E, E)‐2,4‐nonadienal (Bhowmik et al. 2023; Liu et al. 2023); 1‐hexanol and (E)‐2‐heptenal (Bhowmik et al. 2023; Fahmi et al. 2021); (E, E)‐2,4‐heptadienal (Bhowmik et al. 2023); (E)‐2‐octenal (Bhowmik et al. 2023; Fahmi et al. 2021); (E, E)‐3,5‐octadiene‐2‐one, (E)‐2‐decenal, (E, E)‐2,4‐decadienal, and 3‐octen‐2‐one (Liu et al. 2023). Moreover, all identified VOCs showed significant concentration differences between at least two samples. YPI samples in particular exhibited VOC profiles that were consistently distinct from those of other sample types, while YPCs and treated flour samples tended toward greater uniformity.

**TABLE 6:** Concentration (µg/100 g) and odor descriptors of volatile organic compounds (VOCs) identified in yellow pea ingredients and wheat flour.

VOC (µg/100 g)	Odor descriptors^c^	Source of variation	Sample mean values (*n* = 4)^b^					
		(*F* value^a^, 14, 39 df)						
Hexanal	Fresh green fatty aldehydic grassy leafy fruity sweaty	17.88 ***	WF	744.2^de^ (204.7)	YPI‐3	3406.7^ab^ (457.5)	YPI‐8	2699.3^abc^ (341.4)
			YP‐IR	1346.0^cde^ (503.0)	YPI‐4	2287.2^bcd^ (247.5)	YPI‐9	3366.1^ab^ (1343.4)
			YP‐RF	370.5^e^ (14.9)	YPI‐5	2357.7^bcd^ (157.8)	YPC 1	458.2^e^ (107.9)
			YPI‐1	3810.4^ab^ (793.9)	YPI‐6	3395.4^ab^ (868.6)	YPC 2	333.5^e^ (28.0)
			YPI‐2	4139.2^a^ (1414.2)	YPI‐7	2605.0^abc^ (322.5)	YPC 3	373.3^e^ (14.5)
2‐Hexenal	Green banana aldehydic fatty cheesy	19.94 ***	WF	61.9^de^ (7.3)	YPI‐3	264.3^abc^ (45.1)	YPI‐8	188.6^bcd^ (72.9)
			YP‐IR	58.8^de^ (13.2)	YPI‐4	143.1^cde^ (12.1)	YPI‐9	371.0^a^ (92.3)
			YP‐RF	34.5^e^ (1.6)	YPI‐5	143.6^cde^ (15.3)	YPC 1	119.0^de^ (21.6)
			YPI‐1	257.3^abc^ (81.3)	YPI‐6	362.7^a^ (89.4)	YPC 2	76.3^de^ (6.7)
			YPI‐2	261.4^abc^ (70.0)	YPI‐7	311.4^ab^ (27.9)	YPC 3	61.7^de^ (1.8)
1‐Hexanol	Ethereal fusel oily fruity alcoholic sweet green	43.18 ***	WF	166.5^b^ (67.6)	YPI‐3	0.0^d^	YPI‐8	0.0^d^
			YP‐IR	73.4^bcd^ (30.3)	YPI‐4	0.0^d^	YPI‐9	0.0^d^
			YP‐RF	37.6^cd^ (7.8)	YPI‐5	42.8^cd^ (12.5)	YPC 1	575.4^a^ (134.3)
			YPI‐1	0.0^d^	YPI‐6	55.3^bcd^ (7.7)	YPC 2	149.3^bc^ (25.7)
			YPI‐2	0.0^d^	YPI‐7	83.2^bcd^ (11.7)	YPC 3	138.6^bc^ (42.5)
Heptanal	Fresh aldehydic fatty green herbal cognac ozone	7.16 ***	WF	69.3^cd^ (18.0)	YPI‐3	468.4^a^ (318.3)	YPI‐8	139.2^bcd^ (2.2)
			YP‐IR	224.3^abcd^ (81.7)	YPI‐4	322.6^abcd^ (32.2)	YPI‐9	292.3^abcd^ (126.5)
			YP‐RF	68.6^cd^ (5.7)	YPI‐5	296.8^abcd^ (30.0)	YPC 1	70.1^cd^ (12.6)
			YPI‐1	481.5^a^ (110.4)	YPI‐6	362.4^abc^ (76.1)	YPC 2	43.2^d^ (2.8)
			YPI‐2	443.9^ab^ (182.0)	YPI‐7	192.8^abcd^ (7.2)	YPC 3	50.2^d^ (3.9)
2,5‐Dimethyl‐pyrazine	Cocoa roasted nutty beefy roasted beef woody grassy medicinal	68.12 ***	WF	0.0^b^	YPI‐3	0.0^b^	YPI‐8	0.0^b^
			YP‐IR	186.3^a^ (40.2)	YPI‐4	232.3^a^ (30.0)	YPI‐9	224.2^a^ (65.4)
			YP‐RF	50.9^b^ (4.7)	YPI‐5	0.0^b^	YPC 1	0.0^b^
			YPI‐1	0.0^b^	YPI‐6	0.0^b^	YPC 2	0.0^b^
			YPI‐2	0.0^b^	YPI‐7	0.0^b^	YPC 3	0.0^b^
(E)‐2‐Heptenal	Pungent green vegetable fresh fatty	7.74 ***	WF	1259.5^a^ (129.3)	YPI‐3	1118.1^ab^ (745.5)	YPI‐8	672.5^abcd^ (84.4)
			YP‐IR	236.5^cd^ (50.8)	YPI‐4	493.3^bcd^ (89.4)	YPI‐9	1015.3^ab^ (260.2)
			YP‐RF	174.7^d^ (5.9)	YPI‐5	604.1^bcd^ (64.3)	YPC 1	350.8^cd^ (58.6)
			YPI‐1	815.5^abcd^ (63.5)	YPI‐6	689.8^abcd^ (200.2)	YPC 2	208.0^d^ (25.1)
			YPI‐2	868.7^abc^ (257.8)	YPI‐7	679.2^abcd^ (54.2)	YPC 3	253.0^cd^ (22.4)
Benzaldehyde	Sharp sweet bitter almond cherry	71.36 ***	WF	279.9^e^ (37.0)	YPI‐3	10204.6^d^ (6841.2)	YPI‐8	11393.5^d^ (2114.6)
			YP‐IR	1214.8^e^ (316.2)	YPI‐4	12576.8^cd^ (2347.6)	YPI‐9	16441.6^bcd^ (2121.5)
			YP‐RF	804.4^e^ (25.7)	YPI‐5	15142.7^bcd^ (1716.3)	YPC 1	542.6^e^ (142.9)
			YPI‐1	21418.1^b^ (1680.6)	YPI‐6	21047.6^bc^ (4746.6)	YPC 2	270.1^e^ (95.9)
			YPI‐2	51118.9^a^ (6816.4)	YPI‐7	10219.9^d^ (668.2)	YPC 3	624.1^e^ (203.9)
1‐Octen‐3‐ol	Mushroom earthy green oily fungal raw chicken	6.18 ***	WF	425.6^ab^ (150.2)	YPI‐3	661.0^ab^ (449.6)	YPI‐8	148.6^b^ (10.7)
			YP‐IR	388.3^ab^ (92.7)	YPI‐4	487.4^ab^ (82.1)	YPI‐9	257.5^b^ (150.6)
			YP‐RF	199.7^b^ (15.7)	YPI‐5	667.4^ab^ (65.6)	YPC 1	289.7^b^ (71.5)
			YPI‐1	913.6^a^ (164.6)	YPI‐6	520.5^ab^ (111.1)	YPC 2	220.3^b^ (49.3)
			YPI‐2	916.7^a^ (456.4)	YPI‐7	186.2^b^ (33.5)	YPC 3	196.9^b^ (82.8)
2,3‐Octanedione	Dill asparagus cilantro herbal aldehydic earthy fatty cortex	4.71 ***	WF	366.2^abcd^ (88.2)	YPI‐3	443.5^abcd^ (297.6)	YPI‐8	328.7^abcd^ (21.4)
			YP‐IR	219.0^bcd^ (46.8)	YPI‐4	346.3^abcd^ (31.6)	YPI‐9	528.0^ab^ (229.4)
			YP‐RF	133.7^d^ (5.2)	YPI‐5	484.2^abc^ (63.0)	YPC 1	330.5^abcd^ (58.5)
			YPI‐1	629.8^a^ (49.0)	YPI‐6	263.8^bcd^ (74.1)	YPC 2	182.8^cd^ (29.8)
			YPI‐2	384.7^abcd^ (179.1)	YPI‐7	332.1^abcd^ (53.4)	YPC 3	215.2^bcd^ (63.8)
Furan‐2‐pentyl	Fruity green earthy beany vegetable metallic	29.93 ***	WF	3168.9^efg^ (522.2)	YPI‐3	10265.4^cdef^ (7531.3)	YPI‐8	11370.1^bcd^ (704.8)
			YP‐IR	1756.0^g^ (441.1)	YPI‐4	18895.4^b^ (2461.6)	YPI‐9	10769.2^bcde^ (3519.9)
			YP‐RF	1280.1^g^ (164.7)	YPI‐5	10140.6^cdef^ (631.4)	YPC 1	3300.7^defg^ (662.5)
			YPI‐1	32712.5^a^ (3145.8)	YPI‐6	12065.0^bc^ (1338.3)	YPC 2	2169.1^fg^ (209.4)
			YPI‐2	13627.0^bc^ (5702.2)	YPI‐7	12766.5^bc^ (1297.4)	YPC 3	2130.1^fg^ (257.5)
Octanal	Aldehydic waxy citrus orange peel green herbal fresh fatty	7.67 ***	WF	91.3^de^ (36.0)	YPI‐3	626.4^ab^ (440.5)	YPI‐8	162.4^cde^ (11.4)
			YP‐IR	353.4^abcde^ (92.9)	YPI‐4	513.6^abc^ (19.4)	YPI‐9	416.7^abcd^ (122.5)
			YP‐RF	155.6^cde^ (17.9)	YPI‐5	336.1^abcde^ (70.4)	YPC 1	0.0^e^
			YPI‐1	683.6^a^ (206.4)	YPI‐6	315.8^abcde^ (49.4)	YPC 2	67.8^de^ (5.3)
			YPI‐2	272.1^bcde^ (96.7)	YPI‐7	245.8^bcde^ (12.8)	YPC 3	72.0^de^ (8.3)
(E, E)‐2,4‐heptadienal	Fatty green oily aldehydic vegetable cinnamon	26.35 ***	WF	428.8^de^ (109.0)	YPI‐3	1303.7^cde^ (903.0)	YPI‐8	2219.3^bc^ (597.0)
			YP‐IR	89.2^e^ (14.7)	YPI‐4	1076.4^cde^ (262.2)	YPI‐9	1725.2^cd^ (629.8)
			YP‐RF	83.8^e^ (5.7)	YPI‐5	1282.9^cde^ (244.2)	YPC 1	166.6^e^ (37.0)
			YPI‐1	1636.6^cd^ (251.0)	YPI‐6	3212.0^b^ (972.9)	YPC 2	80.2^e^ (21.2)
			YPI‐2	4624.6^a^ (937.1)	YPI‐7	1293.1^cde^ (270.9)	YPC 3	125.2^e^ (28.4)
p‐Cymene	Fresh citrus terpenic woody spicy	7.07 ***	WF	22.5^b^ (7.3)	YPI‐3	111.5^a^ (74.5)	YPI‐8	0.0^b^
			YP‐IR	38.9^b^ (15.1)	YPI‐4	0.0^b^	YPI‐9	0.0^b^
			YP‐RF	19.7^b^ (1.7)	YPI‐5	0.0^b^	YPC 1	56.1^ab^ (27.4)
			YPI‐1	0.0^b^	YPI‐6	0.0^b^	YPC 2	23.6^b^ (2.1)
			YPI‐2	0.0^b^	YPI‐7	0.0^b^	YPC 3	32.2^b^ (18.0)
3‐Octene‐2‐one	Earthy spicy herbal sweet mushroom hay blueberry	22.08 ***	WF	218.2^ef^ (22.6)	YPI‐3	806.0^bcde^ (552.6)	YPI‐8	414.3^cdef^ (42.6)
			YP‐IR	36.0^f^ (8.6)	YPI‐4	969.6^bc^ (83.9)	YPI‐9	414.9^cdef^ (95.8)
			YP‐RF	28.2^f^ (1.6)	YPI‐5	517.9^cdef^ (48.1)	YPC 1	107.6^f^ (18.4)
			YPI‐1	1714.2^a^ (241.6)	YPI‐6	822.6^bcd^ (162.6)	YPC 2	45.2^f^ (4.7)
			YPI‐2	1382.4^ab^ (461.5)	YPI‐7	308.9^def^ (28.5)	YPC 3	44.9^f^ (3.6)
(E)‐2‐octenal	Fresh cucumber fatty green herbal banana waxy green leafy	17.76 ***	WF	520.1^cdef^ (18.1)	YPI‐3	666.6^bcde^ (462.7)	YPI‐8	672.0^bcd^ (78.9)
			YP‐IR	130.8^f^ (29.2)	YPI‐4	575.8^bcdef^ (89.6)	YPI‐9	1317.8^a^ (326.8)
			YP‐RF	127.4^f^ (6.4)	YPI‐5	600.1^bcdef^ (80.7)	YPC 1	245.5^def^ (50.5)
			YPI‐1	924.2^abc^ (99.4)	YPI‐6	1075.1^ab^ (267.5)	YPC 2	142.1^f^ (23.8)
			YPI‐2	1395.6^a^ (318.2)	YPI‐7	925.5^abc^ (80.6)	YPC 3	150.9^ef^ (42.8)
Acetophenone	Sweet pungent hawthorn mimosa almond acacia chemical	26.02 ***	WF	46.4^f^ (7.9)	YPI‐3	899.9^cde^ (623.8)	YPI‐8	1096.5^bcd^ (188.2)
			YP‐IR	55.9^f^ (17.7)	YPI‐4	1067.0^bcd^ (290.8)	YPI‐9	1165.0^bcd^ (156.8)
			YP‐RF	44.6^f^ (6.0)	YPI‐5	1327.1^bc^ (261.6)	YPC 1	428.8^def^ (74.7)
			YPI‐1	2528.2^a^ (561.3)	YPI‐6	1353.5^bc^ (283.8)	YPC 2	237.2^ef^ (43.6)
			YPI‐2	1821.0^ab^ (301.0)	YPI‐7	257.1^ef^ (19.4)	YPC 3	155.0^ef^ (38.6)
(E, E)‐3,5‐octadiene‐2‐one	Fruity green grassy	86.33 ***	WF	133.7^d^ (18.0)	YPI‐3	1341.4^cd^ (987.5)	YPI‐8	1990.1^cd^ (179.5)
			YP‐IR	79.4^d^ (9.4)	YPI‐4	5513.8^b^ (691.1)	YPI‐9	2279.3^c^ (112.1)
			YP‐RF	71.3^d^ (4.1)	YPI‐5	6545.3^b^ (702.6)	YPC 1	314.5^cd^ (60.3)
			YPI‐1	10483.8^a^ (1577.6)	YPI‐6	9019.4^a^ (1685.9)	YPC 2	133.8^d^ (13.3)
			YPI‐2	8908.0^a^ (1257.2)	YPI‐7	1913.6^cd^ (189.4)	YPC 3	116.3^d^ (5.3)
Unknown 1	N/A	85.51 ***	WF	270.6^fg^ (41.7)	YPI‐3	6350.1^efg^ (4388.9)	YPI‐8	11513.2^de^ (1900.5)
			YP‐IR	156.2^g^ (28.4)	YPI‐4	19098.4^cd^ (3157.3)	YPI‐9	10824.0^def^ (1307.2)
			YP‐RF	159.0^g^ (9.4)	YPI‐5	25438.0^bc^ (3728.7)	YPC 1	898.5^fg^ (179.0)
			YPI‐1	33082.7^b^ (3688.7)	YPI‐6	49245.0^a^ (9259.3)	YPC 2	389.7^fg^ (55.4)
			YPI‐2	58570.4^a^ (8901.2)	YPI‐7	9218.8^defg^ (349.8)	YPC 3	301.8^fg^ (93.4)
Nonanal	Waxy aldehydic rose fresh orris orange peel fatty peely	10.23 ***	WF	369.4^f^ (63.9)	YPI‐3	1669.1^abc^ (1113.4)	YPI‐8	972.9^bcdef^ (7.9)
			YP‐IR	1077.5^bcdef^ (214.2)	YPI‐4	1362.4^abcde^ (111.2)	YPI‐9	1523.5^abcd^ (326.8)
			YP‐RF	718.1^def^ (34.9)	YPI‐5	1865.5^ab^ (121.3)	YPC 1	500.0^ef^ (61.4)
			YPI‐1	2242.6^a^ (114.6)	YPI‐6	1024.4^bcdef^ (213.6)	YPC 2	364.1^f^ (42.3)
			YPI‐2	861.8^cdef^ (247.3)	YPI‐7	1088.7^bcdef^ (124.2)	YPC 3	558.1^ef^ (9.4)
(E, E)‐2,4‐octadienal	Green fatty pear melon peely	26.04 ***	WF	67.0^cde^ (9.1)	YPI‐3	115.4^cde^ (85.6)	YPI‐8	169.9^bc^ (41.0)
			YP‐IR	20.3^e^ (4.9)	YPI‐4	96.8^cde^ (16.9)	YPI‐9	244.2^b^ (100.6)
			YP‐RF	20.4^e^ (3.1)	YPI‐5	112.0^cde^ (26.7)	YPC 1	36.7^de^ (11.3)
			YPI‐1	165.7^bc^ (29.1)	YPI‐6	252.2^b^ (55.6)	YPC 2	15.1^e^ (8.3)
			YPI‐2	440.5^a^ (77.0)	YPI‐7	146.8^bcd^ (8.1)	YPC 3	14.4^e^ (8.3)
Unknown 2	N/A	9.70 ***	WF	4.6^cd^ (0.6)	YPI‐3	55.4^a^ (38.6)	YPI‐8	0.0^d^
			YP‐IR	13.1^bcd^ (2.2)	YPI‐4	11.6^bcd^ (0.9)	YPI‐9	57.7^a^ (18.2)
			YP‐RF	12.6^bcd^ (0.6)	YPI‐5	0.0^d^	YPC 1	34.6^abc^ (7.7)
			YPI‐1	22.5^bcd^ (2.2)	YPI‐6	12.2^bcd^ (5.0)	YPC 2	39.3^ab^ (3.9)
			YPI‐2	0.0^d^	YPI‐7	0.0^d^	YPC 3	29.2^abcd^ (2.1)
Unknown 3	N/A	8.96 ***	WF	5.5^de^ (3.5)	YPI‐3	88.9^a^ (61.3)	YPI‐8	0.0^e^
			YP‐IR	24.6^cde^ (5.2)	YPI‐4	14.1^cde^ (2.3)	YPI‐9	77.7^ab^ (26.2)
			YP‐RF	20.1^cde^ (1.6)	YPI‐5	0.0^e^	YPC 1	51.2^abcd^ (11.5)
			YPI‐1	28.3^bcde^ (1.9)	YPI‐6	11.0^cde^ (4.0)	YPC 2	59.3^abc^ (7.3)
			YPI‐2	0.0^e^	YPI‐7	0.0^e^	YPC 3	43.6^abcde^ (4.0)
1H‐indene, 2,3‐dihydro‐4‐methyl	N/A	12.23 ***	WF	3.8^d^ (0.4)	YPI‐3	36.8^ab^ (25.1)	YPI‐8	17.6^bcd^ (2.4)
			YP‐IR	6.3^d^ (1.5)	YPI‐4	0.0^d^	YPI‐9	41.3^a^ (13.8)
			YP‐RF	7.1^cd^ (0.6)	YPI‐5	0.0^d^	YPC 1	32.1^ab^ (9.6)
			YPI‐1	0.0^d^	YPI‐6	0.0^d^	YPC 2	28.5^abc^ (3.8)
			YPI‐2	0.0^d^	YPI‐7	0.0^d^	YPC 3	20.3^abcd^ (0.9)
3‐Nonen 2‐one	Fruity berry fatty oily ketonic weedy spicy licorice	32.71 ***	WF	21.1^e^ (5.1)	YPI‐3	146.7^de^ (101.5)	YPI‐8	99.3^e^ (20.6)
			YP‐IR	2.3^e^ (1.0)	YPI‐4	371.5^bc^ (58.0)	YPI‐9	137.3^de^ (62.0)
			YP‐RF	4.1^e^ (0.7)	YPI‐5	80.3^e^ (13.4)	YPC 1	23.8^e^ (6.2)
			YPI‐1	535.8^ab^ (100.4)	YPI‐6	293.4^cd^ (53.9)	YPC 2	11.0^e^ (1.6)
			YPI‐2	630.6^a^ (184.6)	YPI‐7	89.4^e^ (3.7)	YPC 3	7.5^e^ (6.7)
Unknown 4	N/A	12.86 ***	WF	5.8^e^ (0.4)	YPI‐3	56.5^b^ (39.5)	YPI‐8	42.4^bcd^ (3.0)
			YP‐IR	11.4^de^ (3.0)	YPI‐4	11.6^de^ (2.5)	YPI‐9	96.8^a^ (25.4)
			YP‐RF	11.9^de^ (1.0)	YPI‐5	11.1^de^ (1.5)	YPC 1	42.2^bcd^ (12.0)
			YPI‐1	19.6^cde^ (3.2)	YPI‐6	15.5^cde^ (7.1)	YPC 2	51.3^bc^ (7.2)
			YPI‐2	9.8^de^ (1.5)	YPI‐7	0.0^e^	YPC 3	33.1^bcde^ (5.0)
Citronellal	Sweet dry floral herbal waxy aldehydic citrus	12.43 ***	WF	31.1^c^ (5.6)	YPI‐3	69.8^bc^ (48.4)	YPI‐8	49.1^bc^ (2.8)
			YP‐IR	61.1^bc^ (16.8)	YPI‐4	35.2^c^ (4.6)	YPI‐9	63.2^bc^ (27.7)
			YP‐RF	48.1^bc^ (2.4)	YPI‐5	71.8^bc^ (10.0)	YPC 1	56.5^bc^ (11.3)
			YPI‐1	50.2^bc^ (9.6)	YPI‐6	58.5^bc^ (15.0)	YPC 2	194.2^a^ (34.6)
			YPI‐2	95.4^b^ (18.7)	YPI‐7	46.8^bc^ (5.2)	YPC 3	65.5^bc^ (14.2)
(E)‐2‐nonenal	Fatty green cucumber aldehydic citrus	35.31 ***	WF	176.9^def^ (24.3)	YPI‐3	195.3^def^ (144.2)	YPI‐8	302.3^cde^ (36.6)
			YP‐IR	124.2^ef^ (26.2)	YPI‐4	0.0^f^	YPI‐9	428.7^bc^ (143.4)
			YP‐RF	154.2^def^ (9.8)	YPI‐5	346.9^cd^ (55.7)	YPC 1	0.0^f^
			YPI‐1	0.0^f^	YPI‐6	591.4^ab^ (144.4)	YPC 2	168.1^def^ (43.5)
			YPI‐2	790.8^a^ (130.2)	YPI‐7	295.2^cde^ (8.7)	YPC 3	0.0^f^
2‐Decanone	Orange floral fatty peach	77.86 ***	WF	0.0^f^	YPI‐3	245.0^ef^ (169.0)	YPI‐8	732.7^de^ (7.8)
			YP‐IR	30.5^f^ (14.1)	YPI‐4	1768.0^bc^ (224.6)	YPI‐9	1089.5^d^ (355.3)
			YP‐RF	33.3^f^ (3.6)	YPI‐5	1172.2^cd^ (139.5)	YPC 1	91.1^f^ (23.3)
			YPI‐1	3125.5^a^ (395.7)	YPI‐6	2999.2^a^ (369.5)	YPC 2	92.9^f^ (10.1)
			YPI‐2	2048.9^b^ (517.1)	YPI‐7	1125.7^d^ (111.7)	YPC 3	78.4^f^ (14.4)
Octanoic acid ethyl ester	Fruity winey waxy sweet apricot banana brandy pear	9.27 ***	WF	0.0^c^	YPI‐3	458.2^a^ (307.5)	YPI‐8	153.8^bc^ (16.6)
			YP‐IR	0.0^c^	YPI‐4	0.0^c^	YPI‐9	287.0^ab^ (99.0)
			YP‐RF	0.0^c^	YPI‐5	120.6^bc^ (32.2)	YPC 1	340.4^ab^ (81.3)
			YPI‐1	148.2^bc^ (21.6)	YPI‐6	0.0^c^	YPC 2	143.0^bc^ (38.0)
			YPI‐2	0.0^c^	YPI‐7	22.3^c^ (2.7)	YPC 3	94.4^bc^ (22.2)
Decanal	Sweet aldehydic waxy orange peel citrus floral	2.56 *	WF	184.7^ab^ (264.9)	YPI‐3	164.3^ab^ (113.8)	YPI‐8	220.5^ab^ (8.0)
			YP‐IR	134.6^ab^ (71.8)	YPI‐4	170.2^ab^ (21.8)	YPI‐9	314.2^a^ (125.2)
			YP‐RF	84.8^ab^ (7.8)	YPI‐5	226.3^ab^ (57.7)	YPC 1	58.9^b^ (6.5)
			YPI‐1	252.4^ab^ (49.6)	YPI‐6	167.4^ab^ (55.3)	YPC 2	46.5^b^ (18.3)
			YPI‐2	192.7^ab^ (24.3)	YPI‐7	265.2^ab^ (11.1)	YPC 3	71.5^ab^ (21.9)
(E, E)‐2,4‐nonadienal	Fatty melon waxy green violet‐leaf cucumber fruit tropical chicken fat	35.80 ***	WF	713.5^cd^ (174.8)	YPI‐3	392.2^d^ (291.7)	YPI‐8	2161.5^b^ (655.3)
			YP‐IR	45.6^d^ (13.2)	YPI‐4	260.1^d^ (175.2)	YPI‐9	1826.8^b^ (706.3)
			YP‐RF	87.8^d^ (2.4)	YPI‐5	444.3^d^ (104.9)	YPC 1	132.0^d^ (34.9)
			YPI‐1	388.6^d^ (158.0)	YPI‐6	2177.4^b^ (672.5)	YPC 2	92.2^d^ (32.8)
			YPI‐2	3946.4^a^ (729.5)	YPI‐7	1478.0^bc^ (370.4)	YPC 3	125.5^d^ (57.1)
Carvone	Minty licorice	140.09 ***	WF	361.1^a^ (50.3)	YPI‐3	6.7^d^ (4.6)	YPI‐8	30.2^cd^ (4.7)
			YP‐IR	0.0^d^	YPI‐4	0.0^d^	YPI‐9	0.0^d^
			YP‐RF	0.0^d^	YPI‐5	0.0^d^	YPC 1	120.8^b^ (24.5)
			YPI‐1	0.0^d^	YPI‐6	69.3^c^ (8.2)	YPC 2	8.5^d^ (1.1)
			YPI‐2	0.0^d^	YPI‐7	8.7^d^ (1.8)	YPC 3	41.9^cd^ (8.9)
(E)‐2‐decenal	Waxy fatty earthy green cilantro mushroom aldehydic fried chicken fat tallow	24.96 ***	WF	107.4^ef^ (30.8)	YPI‐3	229.4^def^ (169.9)	YPI‐8	383.8^cde^ (73.0)
			YP‐IR	18.6^f^ (6.4)	YPI‐4	197.3^def^ (61.6)	YPI‐9	632.9^bc^ (285.0)
			YP‐RF	36.8^ef^ (1.8)	YPI‐5	182.1^def^ (37.1)	YPC 1	84.0^ef^ (23.0)
			YPI‐1	264.1^def^ (73.0)	YPI‐6	1176.0^a^ (392.5)	YPC 2	60.3^ef^ (20.2)
			YPI‐2	975.3^ab^ (125.2)	YPI‐7	493.1^cd^ (79.5)	YPC 3	60.5^ef^ (39.9)
2‐Undecanone	Waxy fruity creamy fatty orris floral	44.17 ***	WF	0.0^b^	YPI‐3	113.6^b^ (81.5)	YPI‐8	235.1^b^ (176.2)
			YP‐IR	0.0^b^	YPI‐4	440.3^a^ (89.7)	YPI‐9	0.0^b^
			YP‐RF	0.0^b^	YPI‐5	0.0^b^	YPC 1	183.2^b^ (39.1)
			YPI‐1	500.8^a^ (127.7)	YPI‐6	0.0^b^	YPC 2	0.0^b^
			YPI‐2	0.0^b^	YPI‐7	0.0^b^	YPC 3	0.0^b^
(E, E)‐2,4‐decadienal	Fried fatty geranium green waxy	58.40 ***	WF	0.0^b^	YPI‐3	0.0^b^	YPI‐8	0.0^b^
			YP‐IR	0.0^b^	YPI‐4	0.0^b^	YPI‐9	0.0^b^
			YP‐RF	0.0^b^	YPI‐5	44.9^b^ (10.8)	YPC 1	0.0^b^
			YPI‐1	0.0^b^	YPI‐6	0.0^b^	YPC 2	0.0^b^
			YPI‐2	1344.4^a^ (270.9)	YPI‐7	0.0^b^	YPC 3	0.0^b^

Abbreviations: WF, wheat flour; YPC, yellow pea concentrate; YPI, yellow pea isolate; YP‐IR, yellow pea infrared; YP‐RF, yellow pea radio frequency.

^a^ NS, not significant; *p* ≥ 0.05; ∗*p* < 0.05, ∗∗∗*p* < 0.001, mean values (followed in brackets by the standard deviation) within the same volatile compound with the same letter are not significantly different when a probability level of 0.05 is applied.

^b^ Except for samples YPI‐6, YPI‐7, YPI‐8, YPI‐9, YPC‐2, and YPC‐3, where *n* = 3.

^c^ Reported descriptors: www.thegoodscentscompany.com.

For instance, hexanal, which has been identified both as a LOX product and a potential contributor toward “grassy” or “green” off‐flavors, differed significantly between different YPIs (2287.2–4139.2 µg/100 g), as well as for all YPIs when compared to YPC samples (333.5–458.2 µg/100 g), while YP‐RF (370.5 µg/100 g) resembled YPCs, and YP‐IR concentration (1346.0 µg/100 g) was intermediate between YPCs and YPIs. Furan‐2‐pentyl had a more than 10‐fold difference observed between its lowest concentration in YP‐RF and the highest in YPI‐1 and YPI‐2, while (E, E)‐2,4‐heptadienal was 57 times more concentrated in YPI‐2 than in the lowest‐YPC sample. It was contrary to expectations that the concentrations for all these potential LOX products would be highest in YPIs, given that YPCs exhibited higher LOX activities. One potential explanation for this discrepancy is that YPI and YPC samples likely have significantly different binding capacities for certain VOCs, owing to the disruption of protein‐based interactions that occurred as part of protein isolate production (Wang and Arntfield 2014).

However, this pattern did not hold for all VOCs, with 1‐hexanol being undetectable in all but three YPI samples (YPI‐5, YPI‐6, and YPI‐7), while being present in all YPCs and both treated flour samples. Benzaldehyde, although not typically linked to off‐flavors in YP materials, exhibited the largest concentration difference among samples (189‐fold). Uniquely, 2,5‐dimethyl‐pyrazine was the only compound for which there was a significant difference between the two treated flours, suggesting that, despite their differing modes of action, these two irradiation methods did indeed produce a very similar set of chemical and sensory properties. In contrast, the high variability observed among the nine YPI samples for many VOCs suggests that slight differences in specific processing methods or product origins could potentially result in markedly different chemical and sensory profiles. It is important to note however that while concentrations of VOCs can contribute toward specific sensory perceptions, it can be difficult to predict how a particular ingredient will perform based on its VOC profile alone (Spence 2021).

#### 3.1.4 Electronic Nose Responses

**FIGURE 1:**
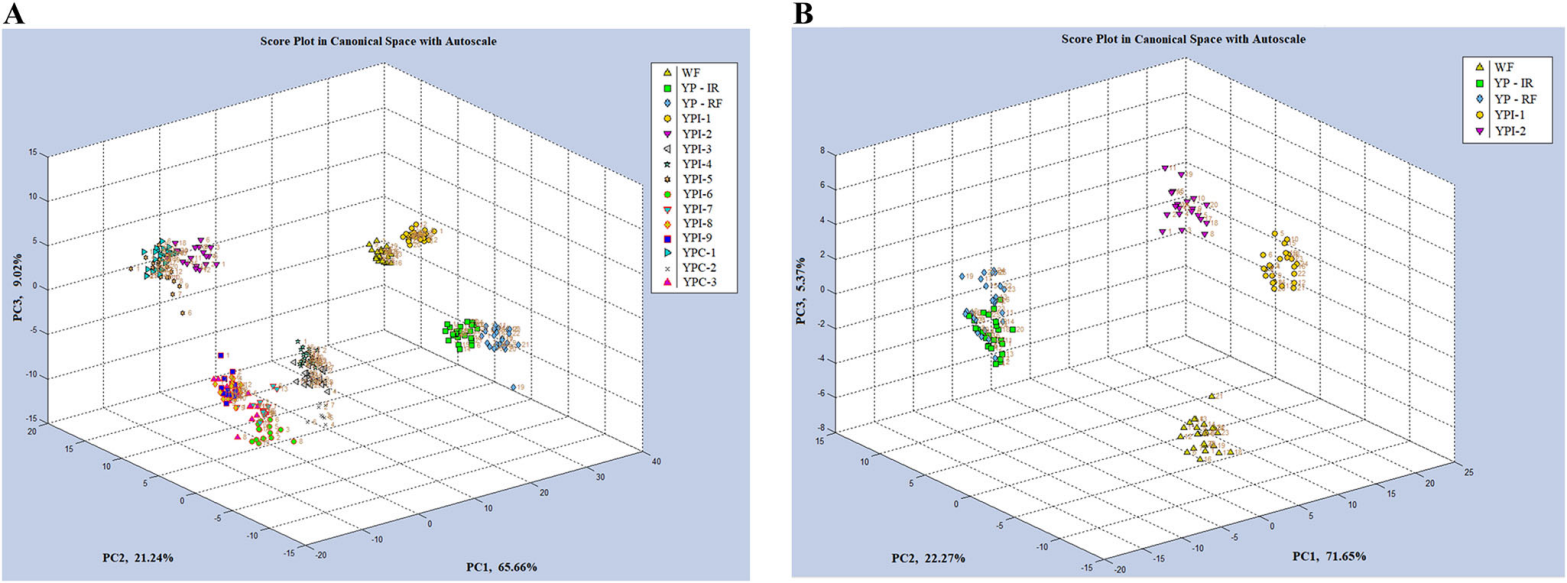
(A) Principal component analysis of eNose response data taken from all yellow pea ingredients and wheat flour. (B) Principal component analysis of eNose response data for selected yellow pea ingredients and wheat flour. WF, wheat flour; YPC, yellow pea concentrate; YPI, yellow pea isolate; YP‐IR, yellow pea infrared; YP‐RF, yellow pea radio frequency.

eNose response data for the YP ingredients and WF are presented in a PCA score plot in Figure 1A. Cross‐validation of this model by CDA correctly classified the samples with a 69.5% level of accuracy. This low prediction rate can be attributed to the grouping of certain samples. For instance, sensor responses for YPI‐2, YPI‐5, and YPC‐1 were largely indistinguishable from one another, while YPI‐9, YPC‐3, YPI‐6, and YPI‐8 formed a second major group, YPI‐3 and YPI‐4 formed a third, and the two treated YP flours formed a fourth. In contrast, YPI‐1, YPC‐2, and WF were all relatively distinct from every other sample in this analysis. While it was expected that the eNose might fail at distinguishing certain samples, it was counterintuitive that two of the four major clusters of sample responses included both YPCs and YPIs, despite these two sample classes possessing very distinct VOC contents. This suggests that the eNose device used may not have been specifically sensitive toward any of the individual VOCs identified, or that differences in sample preparation may have resulted in a distinct set of VOCs being released by the two different experiments (Viana and English 2021).

The eNose response dataset was later reanalyzed using only the four YP ingredients chosen to make bread, as well as WF (Figure 1B). Cross‐validation of the five‐sample model correctly predicted sample identities with an improved 89.0% accuracy rate, with the remaining inaccuracy being attributed to continued overlap between the two treated flours. This result suggested that the WF control bread, and the two YPI breads, would each produce a distinct aroma profile, while the two breads made using treated flours were predicted to be more similar in comparison.

### 3.2 PLS—YP Ingredients

**FIGURE 2:**
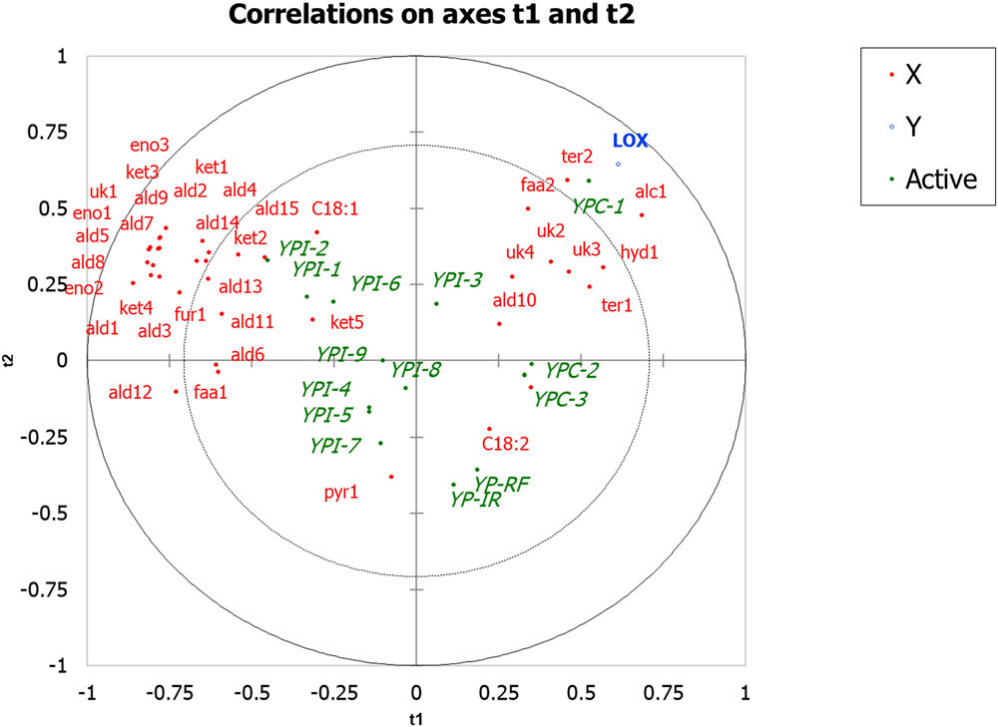
Partial least squares regression (PLS‐R) biplot visualizing relationships among 14 YP ingredients, Y variable (t2): lipoxygenase (LOX) activity; X variables (t1): fatty acid content, volatile organic compounds (VOCs). alc, alcohol; alc1, 1‐hexanol; ald, aldehyde; ald1, hexanal; ald2, 2‐hexenal; ald3, heptanal; ald4, (E)‐2‐heptenal; ald5, benzaldehyde; ald6, octanal; ald7, (E,E)‐2,4‐heptadienal; ald8, (E)‐2‐octenal; ald9, (E,E)‐2,4‐octadienal; ald10, citronellal; ald11, (E)‐2‐nonenal; ald12, decanal; ald13, (E,E)‐2,4‐nonadienal; ald14, (E)‐2‐decenal; ald15, (E,E)‐2,4‐decadienal; eno, enone; eno1, 3‐octene‐2‐one; eno2, (E,E)‐3,5‐octadiene‐2‐one; eno3, 3‐nonen 2‐one; faa, fatty acid; faa1, nonanal; faa2, octanoic acid ethyl ester; fur, furan; fur1, furan‐2‐pentyl; hyd, hydrocarbon; hyd1, 1H‐indene, 2,3‐dihydro‐4‐methyl; ket, ketone; ket1, 1‐octen‐3‐ol; ket2, 2,3‐octanedione; ket3, acetophenone; ket4, 2‐decanone; ket5, 2‐undecanone; pyr, pyrazine; pyr1, 2,5‐dimethyl‐pyrazine; ter, terpenoid; ter1, p‐cymene; ter2, carvone; uk, unknown; uk1, unknown 1; uk2, unknown 2; uk3, unknown 3; uk4, unknown 4; WF, wheat flour; YPC, yellow pea concentrate; YPI, yellow pea isolate; YP‐IR, yellow pea infrared; YP‐RF, yellow pea radio frequency.

A PLS correlation plot (Figure 2) was created using LOX activity as the dependent variable to visualize patterns between samples, selected fatty acids, VOCs, and LOX activity itself. For instance, YPC‐1 was located in the upper right quadrant of this figure, closely associated with LOX activity, along with the off‐flavor compound 1‐hexanol and other VOCs, including p‐cymene, carvone, octanoic acid ethyl ester, and 1H‐Indene, 2,3‐dihydro‐4‐methyl. Based on the assumption that this variable is significantly related to off‐flavor development, samples located in this quadrant were deemed the least suitable for incorporation into bread. Uncorrelated with LOX activity in the bottom‐right quadrant of this figure, the heat‐treated flours, YPC‐2 and YPC‐3 were plotted alongside the nutritionally important fatty acid, and LOX substrate, linoleic acid, and more distantly to the VOCs citronellal and 2,5‐dimethyl‐pyrazine, neither of which were considered off‐flavor compounds. As a result, samples in this quadrant were deemed the most desirable for inclusion into bread for the next phase of this present study. Also orthogonal to LOX activity, samples in the top‐left quadrant were plotted alongside oleic acid and many VOCs associated with “green” or “beany/pea” off‐flavors. Finally, anticorrelated with LOX activity in the bottom left corner were several remaining YPI samples as well as the VOC 2,5‐dimethyl‐pyrazine.

These patterns informed the next part of this study, with two samples being chosen from the bottom‐right quadrant (YP‐IR and YP‐RF) for incorporation into bread based on their predicted favorable sensory and nutritional characteristics. Two samples (YPI‐1, YPI‐2) were also selected from the opposite quadrant based on their predicted levels of high off‐flavors. This choice was further supported by the desire to have at least two samples of a particular type for cross‐comparisons. Slightly more material (20% YP/80% WF) was used in the creation of the YP‐RF‐ and YP‐IR‐enriched breads compared to YPI‐enriched breads (10% YP/90% WF) due to the lower protein content of those ingredients.

#### 3.2.1 Proximate Analysis

Proximate composition of the breads is shown in Table 7. Significant differences were shown between the five bread samples for moisture content, crude protein, fat, ash, carbohydrate, and caloric content. As expected, all YP‐enriched bread contained significantly higher protein compared to the WF sample, with YPI‐1 having significantly higher protein contents compared to YP‐RF. In terms of other effects, moisture was significantly higher in YPI‐2 compared to the WF sample but not significantly different from other YP‐enriched breads. Fat content also varied, with YPI‐2 containing significantly lower fat compared to the other samples. Additionally, the YP‐RF, YP‐IR, and YPI‐1 breads all contained significantly higher ash levels than the WF sample and YPI‐2. Carbohydrate levels were significantly reduced in all YP‐enriched breads, with YPI‐2 having the lowest contents compared to the WF sample. Similarly, YPI‐2 was also significantly less calorically dense (239.33 cal/100 g) compared to WF (272.33 cal/100 g).

**TABLE 7:** Proximate composition and color characteristics of five breads and pH of yellow pea ingredients and wheat flour.

Component	Source of variation (*F* value^a^, 4, 10 df)^b^	Sample mean values (*n* = 3)^c^				
		WF	YP‐IR	YP‐RF	YPI‐1	YPI‐2
Moisture (%)^a^	6.70 **	32.88^b^ (1.44)	36.42^ab^ (3.49)	36.78^ab^ (0.91)	36.53^ab^ (1.38)	40.69^a^ (0.34)
Crude protein (%)^a^	68.00 ***	11.69^c^ (0.25)	15.67^ab^ (0.80)	14.87^b^ (0.17)	16.76^a^ (0.28)	15.70^ab^ (0.10)
Crude fiber (%)^a^	0.53 NS	0.47 (0.13)	0.56 (0.05)	0.55 (0.13)	0.54 (0.15)	0.46 (0.03)
Fat (%)^a^	11.44 ***	1.94^a^ (0.07)	1.93^a^ (0.20)	2.05^a^ (0.04)	1.94^a^ (0.03)	1.55^b^ (0.03)
Ash (%)^a^	13.94 ***	1.38^b^ (0.02)	1.65^a^ (0.09)	1.60^a^ (0.06)	1.61^a^ (0.05)	1.42^b^ (0.03)
Carbohydrate (%)^a^	31.03 ***	52.11^a^ (1.15)	44.33^b^ (2.41)	44.69^b^ (0.70)	43.17^bc^ (1.07)	40.64^c^ (0.29)
Calories (cal/100 g)^a^	7.16 **	272.33^a^ (5.86)	258.33^ab^ (14.47)	256.67^ab^ (3.51)	257.00^ab^ (5.29)	239.33^b^ (1.53)
L* (lightness)^b^	11.04 ***	81.96^a^ (2.50)	76.40^c^ (2.38)	80.88^ab^ (2.79)	77.61^bc^ (3.59)	83.46^a^ (1.81)
a* (green–red color)^b^	1.78 NS	−0.08 (0.49)	0.38 (0.74)	0.31 (0.43)	0.48 (0.53)	0.04 (0.10)
b* (blue–yellow color)^b^	24.54 ***	11.03^d^ (0.64)	14.96^a^ (1.02)	11.94^cd^ (0.67)	13.88^ab^ (0.76)	13.12^bc^ (1.38)
pH	2971.15 ***	6.12^d^ (0.03)	6.54^c^ (0.02)	6.53^c^ (0.01)	6.95^b^ (0.01)	7.18^a^ (0.01)

Abbreviations: WF, wheat flour; YPI, yellow pea isolate; YP‐IR, yellow pea infrared; YP‐RF, yellow pea radio frequency.

^a^ NS, not significant; *p* ≥ 0.05; ∗∗*p* < 0.01, ∗∗∗*p* < 0.001, mean values (followed in brackets by the standard deviation) within the same row with the same letter are not significantly different when a probability level of 0.05 is applied.

^b^ Except for color measurements where (*F* value, 4, 40 df) and pH measurements where (*F* value 4, 25 df).

^c^ Except for color measurements, where *n* = 9 and pH measurements, where *n* = 6.

The compositional shifts may have important implications for bread quality, particularly in terms of texture. For instance, the higher moisture content in YPI‐2 bread could produce softer, more elastic, and “stickier” breads that may more readily adhere to teeth (Jekle and Becker 2012). The increased nonwheat protein contents observed in all YP bread formulations likewise can be expected to disrupt the formation of gluten networks during kneading, potentially resulting in decreased cohesiveness and reduced “springiness” in the final products (Prieto‐Vazquez Del Mercado et al. 2022).

#### 3.2.2 Sensory Analysis

##### 3.2.2.1 Consumer Acceptability

Most of the 65 panelists were female (63%) with age groups represented as follows: 18–24 years—46%; 25–34 years—26%; 35–44 years—11%; 45–54 years—6%; 55–64 years—6%; and over 64 years—5%. Frequency of eating foods that contain mostly YP was at least once per month or more often by 37% of the panelists, 40% less often than once per month, and 23% never. The mean values for acceptability of breads containing YP ingredients ranged from 5.5 “like slightly” to 7.3 “like moderately” compared to the WF control bread that ranged from 6.8 to 7.1 “like moderately” (Table 8). “I like this and would eat it now and then” was the associated mean value for all breads except for YPI‐1, which was “I would eat this if available but would not go out of my way.” Therefore, it was not surprising that YPI‐1 had significantly lower acceptability scores for aroma, color, flavor, overall acceptability, and FACT compared to the WF sample. Meanwhile, YPI‐2, YP‐IR, and YP‐RF bread showed no significant differences for flavor, overall acceptability, and FACT compared to the WF control bread, while YP‐IR bread additionally showed no significant differences in aroma acceptability. Neither texture nor color acceptability showed significant differences between any of the breads, implying that such differences were either not readily apparent or did not influence the acceptability for consumers. This result aligns with a previous study examining bread enriched with heat‐treated YP flours at a 20% level, which found no significant differences in color acceptability when compared to bread with 100% WF (Fahmi et al. 2019).

**TABLE 8:** Consumer acceptability results for breads containing yellow pea ingredients and wheat flour.

	Source of variation (*F* value^a^, 4, 256 df)		Mean acceptability values				
Attribute	Consumer *n* = 65	Sample *n* = 5	WF	YP‐IR	YP‐RF	YPI‐1	YPI‐2
Aroma^b^	3.60 ***	8.28 ***	7.0^a^ (1.6)	6.2^ab^ (1.8)	6.2^b^ (2.0)	5.5^c^ (2.0)	5.9^bc^ (2.0)
Color^b^	7.29 ***	1.50 NS	7.1 (1.6)	7.0 (1.3)	7.1 (1.5)	6.9 (1.4)	7.3 (1.6)
Flavor^b^	3.83 ***	4.80 ***	6.8^a^ (1.5)	6.3^ab^ (1.7)	6.3^ab^ (1.7)	5.7^b^ (2.0)	6.2^ab^ (1.7)
Texture^b^	3.47 ***	0.07 NS	6.8 (1.8)	6.8 (1.6)	6.8 (1.5)	6.9 (1.6)	6.9 (1.8)
Overall acceptability^b^	3.02 ***	3.55 **	6.8^a^ (1.6)	6.4^ab^ (1.6)	6.4^ab^ (1.6)	5.8^b^ (1.9)	6.4^ab^ (1.8)
FACT^c^	3.68 ***	2.89 *	5.9^a^ (1.7)	5.6^ab^ (1.7)	5.6^ab^ (1.7)	5.1^b^ (2.0)	5.5^ab^ (1.8)

Abbreviations: WF, wheat flour; YPI, yellow pea isolate; YP‐IR, yellow pea infrared; YP‐RF, yellow pea radio frequency.

^a^ NS, not significant; *p* ≥ 0.05; ∗*p* < 0.05, ∗∗*p* < 0.01, ∗∗∗*p* < 0.001, mean values (followed in brackets by the standard deviation) within the same row with the same letter are not significantly different when a probability level of 0.05 is applied.

^b^ 1 = dislike extremely; 2 = dislike very much; 3 = dislike moderately; 4 = dislike slightly; 5 = neither like nor dislike; 6 = like slightly; 7 = like moderately; 8 = like very much; 9 = like extremely.

^c^ 1 = I would eat this only if forced; 2 = I would eat this if there were no other food choices; 3 = I would hardly ever eat this; 4 = I don't like this but would eat it on an occasion; 5 = I would eat this if available but would not go out of my way; 6 = I like this and would eat it now and then; 7 = I would frequently eat this; 8 = I would eat this very often; 9 = I would eat this every opportunity I had.

##### 3.2.2.2 Descriptive Analysis

Results for descriptive analysis are shown in Table 9. Replication was found to be significant for chewiness and adhesiveness to teeth, possibly reflecting panelist fatigue or perception drift, while white bread flavor was significant for replication and sample interaction effect, which could imply that some samples were less stable throughout the experiment than others. Significant panelist by replication interactions were found for “wheaty,” “white bread,” “milky,” and “sweet” flavors, as well as for firmness and yellow color. Significant panelist by sample interactions were also observed for “flour,” “white bread,” and “pea” aromas, as well as for “wheaty,” “white bread,” and “pea” flavors, “sour” taste, “springiness,” and “adhesiveness to teeth”; presumably due to panelists’ differences in use of the line scale. Finally, in terms of sample effects, bread samples showed significant differences for all attributes except for “flour” aroma, “wheaty” flavor, “sour” taste, “springiness,” “firmness,” “chewiness,” and “adhesiveness to teeth.”

**TABLE 9:** Descriptive analysis results for breads containing yellow pea ingredients and wheat flour.

Attribute		Source of variation (*F* value^a^, 4, 164 df)						Mean intensity value^b^ (*n* = 33)				
		Sample (S) (*n* = 5)	Panelist (P) (*n* = 11)	Replicate (R) (*n* = 3)	PxR	PxS	RxS	WF	YP‐IR	YP‐RF	YPI‐1	YPI‐2
*Aroma*	Wheaty	3.14 **	28.32 ***	1.87 NS	†	†	†	5.4^ab^ (3.3)	5.7^ab^ (3.4)	5.1^ab^ (3.2)	4.5^b^ (3.0)	6.2^a^ (3.9)
	Sweet	4.72 **	20.42 ***	0.89 NS	†	†	†	4.7^a^ (3.3)	3.6^abc^ (1.8)	3.5^bc^ (2.0)	3.0^c^ (2.1)	4.2^ab^ (3.1)
	Flour	1.93 NS	18.17 ***	1.32 NS	†	2.19 **	†	4.0 (3.0)	3.4 (2.2)	3.5 (2.4)	3.4 (2.5)	4.6 (3.7)
	White bread	4.29 **	5.05 ***	0.75 NS	†	1.68 *	†	7.5^a^ (3.5)	5.8^bc^ (2.8)	5.4^bc^ (3.0)	4.4^c^ (3.2)	6.4^ab^ (3.1)
	Pea	9.33 ***	5.87 ***	0.50 NS	†	2.66 ***	†	3.3^d^ (3.2)	6.9^bc^ (4.3)	7.6^ab^ (4.5)	9.0^a^ (4.1)	5.4^c^ (2.9)
*Flavor/* *Taste*	Wheaty	1.17 NS	17.46 ***	2.70 NS	1.89 *	1.58 *	†	4.6 (3.3)	4.8 (3.3)	4.4 (3.2)	5.0 (3.2)	5.4 (3.5)
	White bread	5.25 **	4.86 ***	1.38 NS	2.05 *	2.43 ***	2.73 *	8.9^a^ (3.1)	7.0^bc^ (3.3)	6.0^cd^ (3.6)	4.7^d^ (2.6)	7.6^ab^ (3.3)
	Milky	15.54 ***	12.26 ***	0.19 NS	2.15 **	†	†	6.6^a^ (2.7)	4.7^bc^ (2.7)	4.5^bc^ (2.7)	3.7^c^ (2.4)	4.8^b^ (2.3)
	Pea	10.13 ***	4.71 ***	2.26 NS	†	3.32 ***	†	2.6^d^ (3.0)	5.8^bc^ (3.6)	7.1^b^ (4.2)	8.6^a^ (4.0)	4.8^c^ (3.1)
	Sweet	8.97 ***	4.45 **	0.69 NS	2.64 **	†	†	5.7^a^ (2.8)	4.9^ab^ (2.4)	4.5^b^ (2.5)	3.1^c^ (2.0)	4.3^bc^ (2.8)
	Sour	2.94 NS	18.48 ***	1.56 NS	†	1.74 *	1.93 NS	2.8 (2.5)	4.1 (3.0)	4.0 (3.2)	5.1 (3.6)	4.7 (4.0)
*Texture*	Springiness	1.21 NS	17.22 ***	0.11 NS	†	1.99 **	†	7.6 (3.5)	8.6 (3.4)	8.1 (3.9)	8.3 (2.6)	8.9 (3.7)
	Firmness	1.57 NS	12.58 ***	0.86 NS	2.03 *	†	†	5.8 (3.0)	5.8 (3.0)	5.9 (2.9)	6.4 (2.8)	6.7 (2.9)
	Denseness	3.20 *	21.27 ***	3.07 NS	†	†	†	6.1^ab^ (3.2)	5.9^b^ (2.8)	6.2^ab^ (3.1)	6.7^ab^ (2.5)	7.4^a^ (2.8)
	Chewiness	1.63 NS	17.29 ***	8.29 ***	†	†	†	7.5 (3.0)	8.3 (3.4)	7.8 (3.2)	7.7 (2.8)	8.6 (2.7)
	Adhesiveness to teeth	0.47 NS	36.58 ***	3.34 *	†	1.56 *	†	7.0 (3.9)	6.8 (4.2)	6.9 (3.6)	6.4 (3.7)	6.5 (3.4)
*Color*	Yellow color	26.96 ***	2.65 *	0.37 NS	5.60 ***	†	†	3.2^c^ (2.2)	6.9^ab^ (3.5)	6.2^b^ (2.8)	7.4^a^ (2.8)	6.3^ab^ (2.7)

Abbreviations: WF, wheat flour; YPI, yellow pea isolate; YP‐IR, yellow pea infrared; YP‐RF, yellow pea radio frequency.

^a^ NS, not significant; *p* ≥ 0.05; ∗*p* < 0.05; ∗∗*p* < 0.01; ∗∗∗*p* < 0.001, mean values (followed in brackets by the standard deviation) within the same attribute (row) with the same letter are not significantly different when a probability level of 0.05 is applied.

^b^ Mean intensity values: 0 (low); 15 (high); † Sums of squares pooled with error as the probability of the interaction effect was ≥ 0.05.

The YPI‐1 breads were found to possess significantly higher “pea” aroma than YPI‐2 and YP‐RF samples, while YPI‐2 possessed significantly stronger “wheaty” aroma in comparison to YPI‐1. Interestingly, the sum of all aroma values was highest in YPI‐2, which could be explained by the fact that this YP ingredient also possessed the highest concentrations for 10 of the 35 VOCs identified, including compounds not solely associated with “beany/pea” or “green” flavors. In contrast to the YPIs, breads made using the two heat‐treated flours showed no significant differences for any aroma attribute between themselves. In terms of flavor attributes, YPI‐1 bread was found to have significantly lower values for “sweet,” “milky,” and “white bread” flavors compared to WF, and significantly higher levels for “pea” flavor. The YPI‐2 bread was not significantly different from the WF control bread in terms of “white bread” flavor, and YP‐IR was not significantly different from the WF control bread in terms of “sweet” taste, while all other samples exhibited decreased values.

Substituting WF with pulse ingredients is known to influence some of the textural properties of breads including decreasing dough stability and increasing the amount of time needed to obtain an optimal gluten network (Prieto‐Vazquez Del Mercado et al. 2022). Within this study, however, only “density” was found to be significantly different between any two bread samples, indicating that the formulations used for the creation of the YP‐enriched breads were effective at recreating the textural characteristics of the control bread. Finally, the “yellow color” for all pea bread samples was significantly higher compared to the WF control bread, and the value for YPI‐1 was significantly higher compared to YP‐RF, though as previously mentioned color was not a significant factor when determining consumer acceptance. Overall, the two breads made with YPI‐1 and YPI‐2 displayed distinct sensory profiles, whereas breads made with the heat‐treated flours fell between the two extremes of the YPIs. These results aligned with predictions obtained from the eNose data, but differed from the PLS model, which suggested that the two YPI ingredients would behave similarly and contrast with the treated flours.

##### 3.2.2.3 Multiple Regression

Multiple regression (MLR) analysis showed that three sensory attributes, springiness, flour aroma, and denseness (descriptive results), were mostly correlated with overall acceptability (consumer results) of the four YP flour‐fortified breads and WF control bread. The standardized beta coefficient values that compare the strength of the effect of individual predictors (independent variable) to overall acceptability in the investigated model were as follows: −0.213 (*p* = 0.005) for “springiness”; 0.258 (*p* = 0.001) for “flour” aroma and −0.222 (*p* = 0.005) for “denseness.” The stepwise model was significant (*F*3,161 = 8.214, *p* = 0.003) with the adjusted *R* ^2^ value of 0.117.

#### 3.2.3 Instrumental Color

Significant differences were noted for both b* (blue–yellow color) and L* (lightness) of the five bread samples (Table 7). Bread made with YP‐IR flour had significantly higher b* values (i.e., more yellow color) than breads with YP‐RF, YPI‐2, and WF, while YP‐RF bread did not differ significantly from the WF control. This result is in contradiction with the results obtained from descriptive sensory analysis, which showed WF control bread as significantly less yellow than all four YP breads. This discrepancy suggests that either the human eye was more sensitive to color differences than the instrument or that the sensory evaluation conditions better highlighted the yellow hue.

In addition, YP‐IR and YPI‐1 had significantly lower L* than the WF control bread and YPI‐2, indicating a darker coloration, while YP1‐2 produced the lightest crumb among YP breads. Previous studies have noted that the addition of protein sources, such as pulse proteins into bread typically darkens the crust and crumb due to enhanced Maillard reactions (Prieto‐Vazquez Del Mercado et al. 2022). Therefore, the mix of both increased and decreased darkness observed here is unusual, particularly for the YP‐IR and YP‐RF breads given their similar behavior in most other analyses. Regardless, color was not a significant factor in determining consumer acceptability, suggesting that these differences are unlikely to hinder the development of YP‐enriched bread formulations.

#### 3.2.4 pH

Results for the pH of the five bread ingredients are presented in Table 7. Of the five samples, the WF sample possessed the lowest pH at 6.12. YP‐RF and YP‐IR flours both had significantly higher pH levels 6.53 and 6.54, respectively, while the pH of YPI‐1 was significantly higher compared to both the WF and the treated flour samples, and YPI‐2 had a significantly higher pH compared to all other samples. These differences have important implications for bread texture, as higher pH values within this range are known to influence dough rheology by increasing the resistance of the material to deformation (Jekle and Becker 2012), which may help to explain the increased “denseness” observed in YPI‐2 bread.

#### 3.2.5 PLS—Breads

**FIGURE 3:**
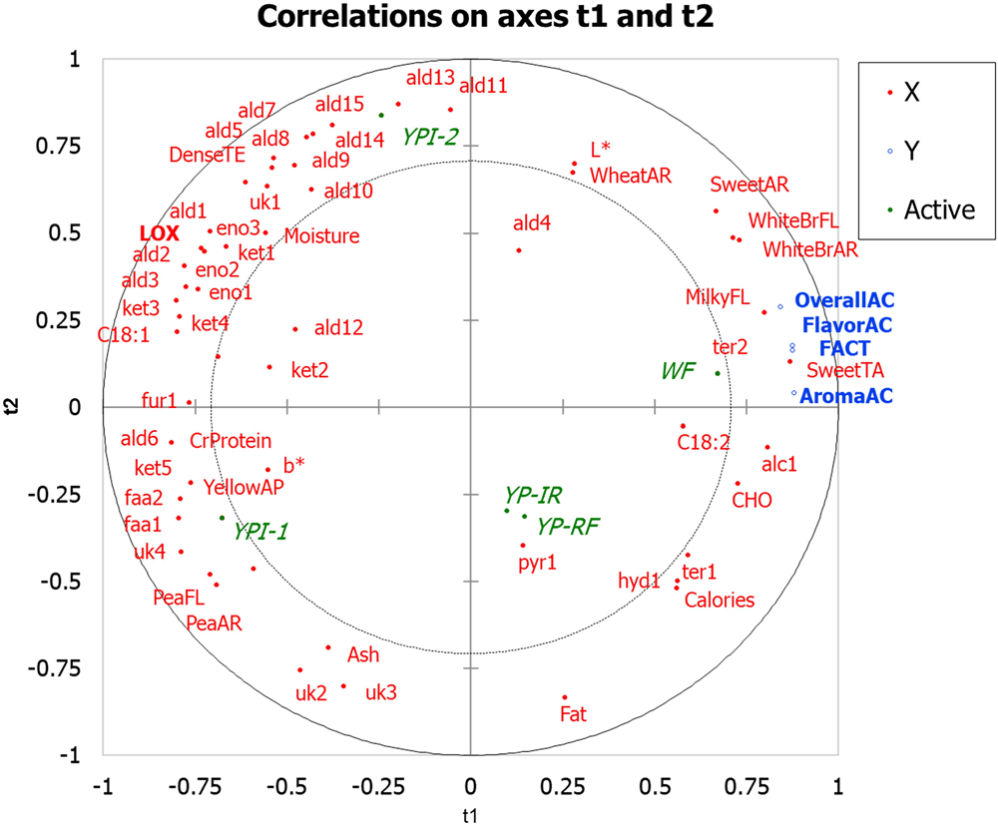
Partial least squares regression (PLS‐R) biplot visualizing relationships for YP‐enriched bread samples. Y variables (t2): sensory acceptance ratings including aroma acceptance, flavor acceptance, overall acceptance, and food action (FACT); X variables (t1): descriptive sensory attributes and instrumental measures including lipoxygenase (LOX) activity, three fatty acids (FAs), and volatile organic compounds (VOCs). acceptability (AC), flavorAC, aromaAC, overallAC; alc, alcohol; alc1, 1‐hexanol; ald, aldehyde; ald1, hexanal; ald2, 2‐hexenal; ald3, heptanal; ald4, (E)‐2‐heptenal; ald5, benzaldehyde; ald6, octanal; ald7, (E,E)‐2,4‐heptadienal; ald8, (E)‐2‐octenal; ald9, (E,E)‐2,4‐octadienal; ald10, citronellal; ald11, (E)‐2‐nonenal; ald12, decanal; ald13, (E,E)‐2,4‐nonadienal; ald14, (E)‐2‐decenal; ald15, (E,E)‐2,4‐decadienal; CHO, carbohydrate, crude fiber, fat, ash, calories (cal/100 g); color (L*, a*, and b*); DenseTE, denseness; eno, enone; eno1, 3‐octene‐2‐one; eno2, (E,E)‐3,5‐octadiene‐2‐one; eno3, 3‐nonen 2‐one; faa, fatty acid; faa1, nonanal; faa2, octanoic acid ethyl ester; FACT, food action rating scale; fur, furan; fur1, furan‐2‐pentyl; hyd, hydrocarbon; hyd1, 1H‐indene, 2,3‐dihydro‐4‐methyl; ket, ketone; ket1, 1‐octen‐3‐ol; ket2, 2,3‐octanedione; ket3, acetophenone; ket4, 2‐decanone; ket5, 2‐undecanone; MilkyFL, milky flavor; PeaAR, pea aroma; proximate composition, crude protein; pyr, pyrazine; PeaFL, pea flavor; pyr1, 2,5‐dimethyl‐pyrazine; SweetAR, sweet aroma; SweetTA, sweet taste; ter, terpenoid; ter1, p‐cymene; ter2, carvone; uk, unknown; uk1, unknown 1; uk2, unknown 2; uk3, unknown 3; uk4, unknown 4; WF, wheat flour; wheatyAR, wheaty aroma; WhiteBrAR, white bread aroma; WhiteBrFL, white bread flavor; YellowAP, yellow color; YPI, yellow pea isolate; YP‐IR, yellow pea infrared; YP‐RF, yellow pea radio frequency.

A second PLS biplot (Figure 3) was generated to explore relationships between statistically significant sensory and instrumental variables for the YP‐enriched breads and their respective ingredients, using consumer acceptability measures as the primary response variables. The WF control bread located in the upper right quadrant clustered closely with the consumer acceptability variables, as well as “milky” flavor, “sweet” taste, and aroma, and “white bread” flavor and aroma, indicating that these characteristics are important drivers of consumer acceptance. Among the YP breads, YP‐IR and YP‐RF breads came closest to this cluster of variables, while being more strongly aligned with total fat, caloric content, carbohydrate, and 2,5‐dimethyl‐pyrazine a VOC which was not associated with YP off‐flavors. The two YPI breads were positioned on opposite ends of the left half of the diagram, each linked with a unique set of variables. YPI‐1 bread was negatively correlated with acceptability, and associated with yellow appearance, pea flavor and aroma, crude protein, ash, and several VOCs including 2‐undecanone, octanal, nonanal, and octanoic acid ethyl ester, although none of these VOCs are recognized as contributors to “beany/pea” flavors. YPI‐2 bread was associated with “denseness,” higher pH, as well as the VOCs benzaldehyde, (E, E)‐2,4‐heptadienal, (E)‐2‐octenal, (E, E)‐2,4‐octadienal, decanal, (E, E)‐2,4‐nonadienal, (E)‐2‐decenal, (E, E)‐2,4‐decadienal, and citronellal. Moisture, LOX activity, and the off‐flavor VOCs hexanal, heptanal, and furan‐2‐pentyl were positioned equidistant between the two YPI breads.

## 4 Conclusion

In the initial stage of this study, 14 YP ingredients were evaluated in terms of their fatty acid concentrations, LOX activity, VOC profiles, and eNose responses to predict their performance in bread formulations. While some variability was noted for YPIs, fatty acid compositions were consistent with those of previously reported YP materials and each other. Class‐based differences were evident for LOX activity, with two of the three YPCs possessing higher levels compared to heat‐treated flours and YPIs. The VOC profiles were also largely determined by processing type, with YPI samples possessing higher levels of many VOCs compared to other sample types, with YPI‐2 having the highest levels of many VOCs. eNose analysis further distinguished between ingredient types, clearly separating samples such as YPI‐1 and YPI‐2, while grouping the heat‐treated flours together. Based on these results, two sets of two samples showing considerable contrast uncorrelated with LOX activity were identified as suitable for inclusion into bread.

In the subsequent bread formulation and evaluation stages, incorporation of the selected YP ingredients into bread formulations altered the nutritional composition of the breads relative to the WF control, with YP‐enriched breads showing elevated protein levels, reduced carbohydrates, and varied moisture, fat, and ash contents. Bread made with YPI‐1 also had a significantly lower acceptability rating compared to bread with 100% WF, as well as higher levels of pea flavor. YPI‐2 bread exhibited a denser texture, which may have been related to the more basic pH of the raw ingredient and an association with “wheat” aroma. Despite YPI‐2's higher VOC content, its sensory acceptability did not differ significantly from YPI‐1, underscoring the complexity of linking specific volatiles to perceived flavor. Extremely few physical and chemical differences were noted between the heat‐treated flours YP‐RF and YP‐IR throughout this study, and these breads behaved most similarly to the control. This indicates that both heating methods were equally suitable for modifying the sensory properties of YP ingredients, despite some initial expectations otherwise.

Beyond these findings, limitations of the present study should be acknowledged. For instance, resources prevented all 14 YP ingredients from being incorporated into bread formulations. While the four samples selected for bread‐making did provide valuable insights, the inclusion of other samples in the bread‐making process, such as the YPCs, as well as keeping the amount of YP ingredient in each bread sample consistent might have revealed additional trends. Including equal amounts of YPI, YPC, and heat‐treated flours in the study would also have helped to clarify the variability between these different classes. Monitoring LOX activity throughout YP ingredient storage, could possibly have explained the discrepancy between relatively high levels of off‐flavor VOCs and low levels of LOX activity present in many YPI samples. An analysis of VOCs in the bread samples themselves may have confirmed the identities of key off‐flavor VOCs between the various samples. This study, however, has shown that heat‐treatment of YP flours produced YP‐enriched breads with acceptable nutritional and sensory properties and highlighted the usefulness of various flavoromics tools as sensory predictors during product development.

## Author Contributions

**Alexandre D. Goertzen**: methodology, writing–original draft, formal analysis, data curation, writing–review and editing. **Donna Ryland**: writing–review and editing, formal analysis, project administration, data curation, supervision. **Shiva Shariati‐Ievari**: formal analysis, data curation, writing–review and editing. **Karen Pitura**: formal analysis, data curation, resources, writing–review and editing. **Lindsay Bourré**: methodology, formal analysis, data curation, resources, writing–review and editing. **Praiya Asavajaru**: resources, writing–review and editing. **Nandhakishore Rajagopalan**: resources, writing–review and editing, methodology. **Anusha G. P. Samaranayaka**: resources, writing–review and editing, methodology. **Brittany Polley**: methodology, data curation, formal analysis, writing–review and editing. **Pankaj Bhowmik**: conceptualization, investigation, funding acquisition, writing–review and editing, methodology, validation, formal analysis, project administration, resources, supervision, data curation. **Michel Aliani**: conceptualization, investigation, funding acquisition, methodology, validation, visualization, writing–review and editing, formal analysis, project administration, data curation, supervision, resources.

## Nomenclature

eNose: electronic nose
FACT: food action rating scale
IR: infrared radiation
LOX: lipoxygenase
PLS: partial least squares
RF: radiofrequency radiation
WF: wheat flour
YP: yellow pea
YPC: YP protein concentrate
YPI: YP protein isolate

## Conflicts of Interest

The authors declare no conflicts of interest.

## Supporting information




**Supporting Information Table S1**: jfds70724‐sup‐0001‐tableS1.docx
