# Supplementary material for: Investigation of Flavor and Functional Properties of Diverse Yellow Pea Ingredients for Pan Bread Applications
Source: J Food Sci. 2025 Dec 11;90(12):e70724. doi: 10.1111/1750-3841.70724 (PMC12699186; doi:10.1111/1750-3841.70724)
Supplement: Supplementary file 1 — Supporting Information Table S1: jfds70724‐sup‐0001‐tableS1.docx [file JFDS-90-0-s001.docx]

**Supplementary Table S1:** The list of identified volatile organic compounds (VOCs) their corresponding quantitative ion, linear retention index, and retention time.

| **VOCs** | **Quantitative**  **Ion** | **Reported**  **LRI^1^** | **Retention**  **Time** |
| --- | --- | --- | --- |
| Hexanal | 56 | 800 | 5.730 |
| 2-Hexenal | 83 | 854 | 7.740 |
| 1-Hexanol | 56 | 868 | 8.440 |
| Heptanal | 70 | 901 | 9.740 |
| 2,5-Dimethyl-pyrazine | 108 | 917 | 9.966 |
| (E)-2-Heptenal | 83 | 958 | 12.000 |
| Benzaldehyde | 108 | 962 | 12.037 |
| 1-Octen-3-ol | 57 | 980 | 13.000 |
| 2,3-Octanedione | 99 | 988 | 13.237 |
| Furan-2-Pentyl | 81 | 993 | 13.500 |
| Octanal | 84 | 1003 | 14.000 |
| (E, E)-2,4-Heptadienal | 81 | 1012 | 14.300 |
| p-Cymene | 119 | 1025 | 14.750 |
| 3-Octene-2-One | 111 | 1040 | 15.425 |
| (E)-2-Octenal | 70 | 1060 | 16.200 |
| Acetophenone | 105 | 1065 | 16.440 |
| (E, E)-3,5-Octadiene 2-one | 95 | 1091 | 16.692 |
| Unknown 1 | 122 | 1099 | 18.029 |
| Nonanal | 57 | 1104 | 18.100 |
| (E, E)-2,4-Octadienal | 81 | 1115 | 18.179 |
| Unknown 2 | 134 | 1125 | 18.620 |
| Unknown 3 | 69 | 1130 | 18.830 |
| 1H-Indene, 2,3-dihydro-4-methyl | 97 | 1136 | 19.056 |
| 3-Nonen 2-one | 125 | 1142 | 19.361 |
| Unknown 4 | 133 | 1147 | 19.453 |
| Citronellal | 69 | 1153 | 19.840 |
| (E)-2-Nonenal | 70 | 1162 | 20.102 |
| 2-Decanone | 58 | 1193 | 21.363 |
| Octanoic acid ethyl ester | 88 | 1198 | 21.557 |
| Decanal | 57 | 1206 | 21.813 |
| (E, E)-2,4-Nonadienal | 81 | 1216 | 22.100 |
| Carvone | 82 | 1242 | 23.140 |
| (E)-2-Decenal | 70 | 1263 | 23.780 |
| 2-Undecanone | 58 | 1277 | 24.153 |
| (E, E)-2,4- Decadienal | 81 | 1317 | 24.891 |

^1^Linear retention index (LRI) from National Institute of Standards and Technology (version 2.3, 2017)
